# Morphometrics of the preserved post-surgical hemisphere in paediatric drug-resistant epilepsy

**DOI:** 10.1101/2023.09.24.559189

**Published:** 2023-09-25

**Authors:** Michael C. Granovetter, Anne Margarette S. Maallo, Christina Patterson, Daniel Glen, Marlene Behrmann

**Affiliations:** 1 Department of Psychology and Neuroscience Institute, Carnegie Mellon University, Pittsburgh, PA, USA 15213; 2 School of Medicine, University of Pittsburgh, Pittsburgh, PA, USA 15213; 3 Department of Pediatrics, University of Pittsburgh, Pittsburgh, PA, USA 15213; 4 Scientific and Statistical Computing Core, National Institute of Mental Health, Bethesda, MD, USA 20892; 5 Department of Ophthalmology, University of Pittsburgh, Pittsburgh, PA, USA 15213

**Keywords:** paediatric, epilepsy, surgery, morphometry, thickness, volume, RRID:SCR_023356

## Abstract

Characterization of the postoperative structural integrity of cortex in adults who have undergone cortical resection surgery for the management of epilepsy has yielded mixed findings. In some cases, patients show persistent or accelerated cortical atrophy, while in others, atrophy decelerates or even reverses. Whether this variability applies as well to a paediatric population, for whom postoperative plasticity may be greater, remains to be determined.

In this case-control study, high resolution structural T1 MRI data were acquired from 32 patients with childhood epilepsy surgery and 51 non-neurological matched controls. Using enhanced automated segmentation capabilities of FreeSurfer Software Suite, we quantified morphometrics of the preserved hemisphere at the level of gross anatomy (lateral ventricle size, volume of grey matter and white matter). Additionally, cortical thickness, volume, and surface area were measured for 34 cortical regions segmented based on the Desikan-Killiany atlas, and, last, volumes of nine subcortical regions were also measured. Morphometry comparisons were made between patients’ preserved hemisphere be it left (LH) or right (RH) against the corresponding hemisphere of age-matched, typically developing controls; and then the two patients groups (LH versus RH) were compared.

Patient groups had larger ventricles and reduced total white matter volume relative to controls, and only patients with a preserved RH, but not patients with a preserved LH, had reduced total grey matter volume relative to controls. Furthermore, patients with a preserved RH had lower cortical thickness and cortical volume and significantly greater cortical surface area of several cortical regions, relative to controls. Patients with a preserved LH had largely no differences in thickness, volume, or area, of any of the 34 cortical regions, relative to controls. Moreover, both LH and RH patients showed reduced volumes in select subcortical structures, relative to controls.

That left-sided, but not right-sided, resection is associated with more pronounced reduction in cortical thickness and volume and increased cortical surface area relative to typically developing, age-matched controls suggests that the preserved RH undergoes plastic processes to an extent not observed in cases of right-sided paediatric resection. Given the importance of understanding outcomes following surgery to the LH versus RH, the post-operative characterisation of morphometrics noted here provides a foundation for future work to understand differences in plasticity as a function of the side preserved post-resection. Future work probing the association of the current findings with neuropsychological outcomes will be necessary to understand the implications of these structural findings for clinical practice.

## Introduction

Epilepsy is a complex, progressive neurological disorder characterized by disruption of the normal balance of excitation and inhibition and sudden spikes of electrical discharge, all of which may result in neuronal impairment, axonal damage, and altered neural circuitry.^1^ Pharmacological treatment is often successful for seizure management, but, for 7–20% of paediatric and 30–40% of adult patients with drug-resistant epilepsy (DRE),^2, 3^ seizure control can be achieved by surgical resection of the epileptogenic cortex with seizure freedom in 86% and 81% of patients at 1 and 2 years follow-up, respectively.^4^

Reports on the postoperative structural integrity of non-resected cortex have yielded mixed findings. Post-surgically, persistent and possibly accelerated reduction in cortical thickness (CxT), a proxy for structural integrity of the brain,^5^ has been identified beyond the defined epileptogenic zone/lobe and is evident even in the preserved hemisphere.^6–9^ In contrast, postsurgical halting of progressive cortical atrophy has also been reported,^6, 10–12^ and, in some patients with temporal lobe epilepsy, the pre-surgical reduction in CxT can even be reversed to within normal limits^6^ (for commentary, see McDonald et al.^13^). Importantly, the extent of reversal is correlated with better seizure control,^5, 6, 10^ as well as positive cognitive outcomes.^14^ Furthermore, reversed cortical volume (CV) loss has also been observed at younger ages, albeit in a small sample size limited to patients with anterior temporal lobectomy.^15^ An explanation of the discrepant atrophy versus recovery in the postoperative period is elusive, and further studies are warranted. Such studies are particularly important given the growing consensus that surgery is underutilised and should be considered a first-line intervention for DRE.^16–18^ If the surgical outcome is of progressive, rather than reduced atrophy, alternative, disease-modifying treatments might be needed to halt further progression.

Here, we compared the postsurgical morphometry of the preserved hemisphere of 32 children with DRE and 51 age-matched typically developing controls on (i) gross anatomical measures of the lateral ventricles (LV), grey matter (GM), and white matter (WM); (ii) CxT (the average distance between the white and pial surfaces), CV, and cortical surface area (CSA) separately for 34 cortical parcels; and (iii) volume of nine subcortical structures. CxT, CV, and CSA in particular are commonly utilised in studying DRE;^19^ have distinct genetic profiles, life span trajectories, and associations with disease;^20–22^ and are differentially associated with cognitive development and neurodevelopmental disorders.^23–25^ Whereas CxT development follows a linear decrease with age,^26^ CSA and CV follow a curvilinear trajectory with CV peaking earlier than CSA.^21^ The complementary information gleaned from these measures offers unique insights to any structural perturbations. Lastly, although postsurgical reduction in cortical thinning affects both hemispheres equally in adults,^10^ here we determined for this paediatric sample, whether any postsurgical structural differences depended on the preserved hemisphere’s being left or right. We focus our analyses here on the preserved hemisphere, as the hemisphere ipsilesional to surgery may have structural abnormalities directly related to the surgery itself, which confounds any postoperative plasticity that we hypothesise occurs post-*paediatric* resection.

We employed general linear modeling (GLM) to compare patients vs. controls and patients’ preserved LH vs. patients’ preserved RH. We also adopted multivariate regression to reveal a model that uses structural parameters to predict group assignment and, in the patients, seizure burden using the International League Against Epilepsy (ILAE) outcome classification schema.^27^

## Materials and methods

### Participants

Thirty-two paediatric patients with DRE and subsequent cortical resection or ablation were recruited for this study. There were 13 patients with LH surgery and a preserved RH (median age/median absolute deviation of age: 15.7/1.7 yr; 6 females, 7 males) and 19 patients with RH surgery and a preserved LH (median age/median absolute deviation of age: 15.4/3.7 yr; 11 females, 8 males). The patients’ demographic information and medical history are detailed in Table 1. Note that we use “RH patient” to refer to patients with left-sided resections but a preserved RH, as it is the RH from which we derive the measures, and the same holds true for “LH patient” in reference to the preserved LH of these patients.

The two patient groups were matched on age (*F*(1,30)=1.12; *p*=.30), gender (*z*(30)=0.65; *p*=.51), age at first surgery (*F*(1,30)=0.03; *p*=.87), seizure-onset age (*F*(1,29)=0.01; *p*=0.93), and binned ILAE outcome scales^28^ (*z*(30)=1.29; *p*=.20). Most patients underwent surgery at University of Pittsburgh Medical Center Children’s Hospital of Pittsburgh, and additional patients were recruited with the assistance of the Pediatric Epilepsy Surgery Alliance. Given the rarity of this patient population, data were not collected prospectively; retrospective analyses were conducted on available control and patient scans from 2013 to 2022.

The 51 control participants (median age/median deviation of age: 14.8/4.9 yr; 24 females, 27 males) did not differ from the patients in age, either in aggregate, (*F*(1,81)=1.18; *p*=.28) or by hemisphere (LH: *F*(1,68)=0.17; *p*=.69 | RH: *F*(1,62)=1.88; *p*=.18) and were matched to the patients on gender (aggregate: *z*(81)=0.54; *p*=.59 | LH: *z*(68)=0.80; *p*=.42 | RH: *z*(62)=0.06; *p*=.95). All participants received $75 per hour for their participation. Where possible, participants signed assent or consent, if appropriate, and guardians consented to a protocol approved by the Institutional Review Boards of Carnegie Mellon University (CMU) and the University of Pittsburgh.

### MRI Protocol

T1-weighted images were acquired using a MPRAGE sequence (1 mm isotropic resolution, TE=1.97 ms, TR=2300 ms, total scan time≅5 min). The images were obtained from either a Siemens Verio 3T scanner with a 32-channel head coil at CMU (36 controls, 13 patients) or a Siemens Prisma 3T scanner with a 64-channel head coil at the CMU-Pitt BRIDGE Center (RRID:SCR_023356; 15 controls, 19 patients).

### Outcome Measures

The anatomical images were preprocessed (motion correction, intensity normalization, and skull stripping), then segmented with the recon-all pipeline of FreeSurfer (v7.1.0),^29–31^ and, finally, the output was manually inspected. As the recon-all pipeline does not generally result in accurate segmentation of hemispherectomy/hemispherotomy/hemidecortication patients, in these patients, the intact hemisphere was mirrored using affine and non-linear transformations (lesion_align program in AFNI) previously shown robust to anatomical aberrations.^32, 33^ Only the data from the actual non-resected hemisphere were analyzed.

Morphometric measures were derived from the preserved hemisphere of the patients and, separately, from each hemisphere of the controls, as follows: (i) *gross measures* of volume of GM, WM, and LV; (ii) cortical morphometry for 34 regions of interest, parcellated according to the Desikan-Killiany cortical atlas;^36, 37^; and (iii) *subcortical structures*^34, 35^ with the volume of nine regions, each normalized to total hemisphere volume (sum of GM, WM, and LV volumes). For the cortical morphometry, for each participant, we calculate CxT in mm, which we did not normalize (as per Westman et al.^38^); CSA in mm^2^ by taking the area of each region and dividing it by the mean of all of the regions in that hemisphere only; and CV in mm^3^ normalized by total hemisphere volume.^39–41^

### Statistical Analysis

Data were analyzed in R^42^ (for packages utilized, see Supplemental Table 1) and SPSS 29.0.1.0. To harmonize data across the two MRI machines, ComBat^43^ was used to model the data as a linear combination of group, age, gender, and scanner, with the assumption that scanner effects have both additive and multiplicative factors.^44^ Furthermore, for each measure and ROI, data were winsorized so as not to lose any data: values above the 95th and below the 5th percentile of the distribution were replaced with an approximation of the corresponding percentile values (separately for controls and patients, and by hemisphere).

For each dependent measure, using GLM, the intact LH or RH of the patients was compared to the controls’ corresponding LH or RH, and then the two patient groups were compared against each other with hemisphere as a between-subjects factor. To avoid concern for committing Type 1 errors with multiple comparisons, we applied specified methods for multiplicity of testing with family-wise correction on all the *p*-values.

Given the relatively small sample sizes in all analyses, for each dependent measure, permutation testing was implemented by randomly shuffling the group label, and a GLM was then fit with group as the primary predictor of interest and age and gender as covariates. This was repeated 1,000 times to create a distribution of the β-coefficients for the effect of group. A *p*-value was then calculated as the percentage of occurrences in which the absolute value of the β-coefficient from the simulated distribution exceeded the absolute value of the true β-coefficient. Significance was ascertained at an α-criterion of .05, and, per measure, the Benjamini-Hochberg correction^45^ was applied to *p*-values across the total number of ROIs. In addition, for further adjudication of the findings, Bayes factor (BF) was computed by comparing the model with the group term to a null model without the group term.^46, 47^ This was especially important to examine null results and to obtain further evidence to inform the absence of significant effects. BFs below 0.33 and above 3 were interpreted as evidence for the alternative hypothesis and for the null hypothesis, respectively.

The above analyses examined each dependent measure separately between patients and controls and between the two patient subgroups. To elucidate which measures, alone or in combination, predicted group membership for each patient group and the matched hemisphere of the controls and between patient groups, we conducted forward binary logistic regression analyses. Such a procedure yields the variables that account for significant variance, over and above other significant variables already entered into the model. Last, we predicted ILAE outcome (high versus low) for the patients using a multivariate approach. The *r*^*2*^ of the regression models are provided using the well-established Nagelkerke measure (a modification of the Cox and Snell R Square, which is derived from the likelihood ratio test statistic), and the factors entered are terminated when the model change is less than .001. The Nagelkerke values indicate that a value of 0.2 or less indicates a weak relationship between the predictors and the outcome, a value of 0.2 to 0.4 indicates a moderate relationship, and a value of 0.4 or higher indicates a strong relationship. Because we are only interested in values indicating at least a moderate or a strong relationship, we only report those computed *r*^*2*^ values that are greater than 0.25 and, for those below 0.25, we simply report the presence of a weak relationship between the predictors and outcome and do not interpret further.

## Results

For all analyses, the statistical comparisons are reported in the following sequence. First, we report the findings of each GLM, separately, relative to their corresponding matched controls. We then describe the findings from comparisons of the two patient groups against each other. Note that age and gender are included as covariates in every analysis. Thereafter, we report the results of a forward binary logistic regression analysis identifying the factors that best predict group assignment.

### Gross Anatomical Morphometrics

**Figure 1:**
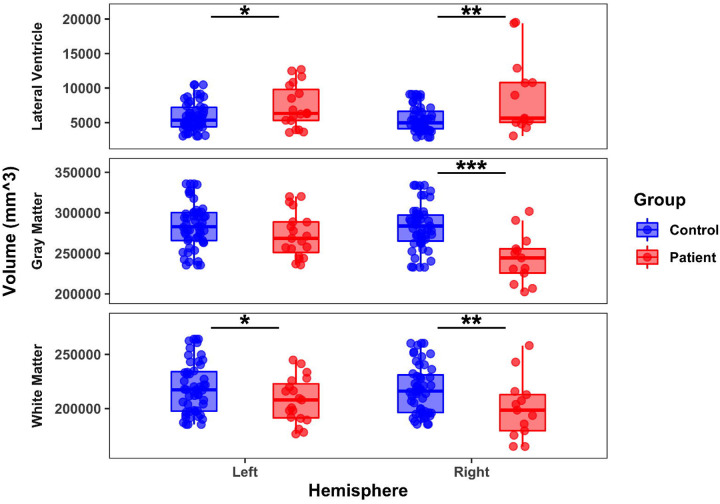
Volume of (top) lateral ventricles, (middle) grey matter, and (bottom) white matter for patients and controls, separately for the preserved left and right hemispheres. *: *p*<.05; **: *p*<.01; ***: *p*<.001. Each dot represents a single participant, and the solid horizontal line in the box indicates the median of the group.

GLM revealed that relative to the LH of the controls, LH patients had significantly larger LV volume (*p*=.02, *BF*=0.57) and significantly smaller WM volume (*p*=.05, *BF*=1.02), and no difference on GM volume (*p*=.27, *BF*=4.37). Relative to the RH of the controls, the RH patient group had significantly larger LV volume (*p*<.01, *BF*=0.01), significantly smaller WM volume (*p*<.01, *BF*=5.56*10^−2^) but also significantly smaller GM volume (*p*<0.001, *BF*=1.6*10^−3^). The volume of the LV did not differ across the two patient groups (*p*=.55, *BF*=4.54), nor did the volume of the WM differ (*p*=.13, *BF*=1.39). However, the LH patient group had significantly greater GM volume than the RH patient group (*p*<.01, *BF*=1.98*10^−2^). The data and results of these analyses are shown in Figure 1.

To evaluate which of these three dependent measures, LV, GM, and WM, singly or in combination predicted group membership, we used forward binary logistic regression with *p* < .05 for entry criterion: LH patients and LH controls could not be reliably discriminated (model with low *r*^*2*^ indicating a weak relationship between the predictors and outcome). Restricting this same analysis to RH patients and RH controls yielded a model of GM and LV, in that order, but not WM, with an *r*^*2*^ of 0.5. GM alone was the best predictor of which hemisphere is preserved in direct comparison of the patient subgroups with a *r*^*2*^ = 0.28. Last, we examined whether these gross anatomical measures might predict seizure burden post-surgery using ILAE scores: no predictor/s were able to differentiate the ILAE binned scores of the patients.

Together, the results of the GLM analyses were well supported by the logistic regression models. Patients with a preserved LH differed from their matched controls on LV and WM, but not GM, but the group differences were not reliable on binary regression analysis. The patients with a preserved RH differed significantly from controls on all three measures of gross anatomy; however, just GM and LV, and not WM, combined into a moderately predictive regression model. GM alone predicted patient group membership across the two patient groups, and ILAE outcome could not be predicted by any dependent measure/s.

### Cortical Regional Morphometrics

This section presents the GLM analyses separately for three dependent measures, CxT in mm, SA in mm^2^, and CV in mm^3^. We then report forward binary regression analyses, as above, separately for the three dependent measures, to determine the model/s that best predict group membership and ILAE binned scale. Last, we conduct a full multivariate regression including all three dependent variables for each of the 34 regions for predicting LH and RH patients against their respective controls and then against each other to determine side of non-resected hemisphere.

### Cortical Thickness

**Figure 2:**
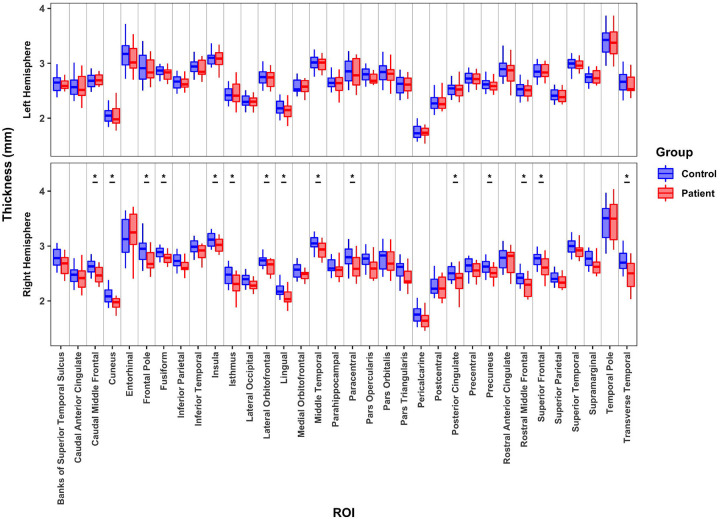
Median cortical thickness for 34 cortical regions for patients with a preserved left hemisphere or right hemisphere relative to their matched controls. *: *p*<0.05; **: *p*<0.01; ***: *p*<0.001.

As shown in Figure 2, relative to the LH controls, the LH patients showed no statistically significant differences in CxT in any of 34 areas whereas, compared to the RH of controls, the RH patients had significantly lower CxT in 15 of the 34 cortical regions and no region with significantly higher thickness. The RH patients had lower cortical CxT in three regions compared with the LH patients, namely, the caudal middle frontal, rostral middle frontal, and superior frontal regions. (See Supplementary Table 2 for individual *p* and *BF* values.)

A forward regression analysis with CxT indices of all 34 regions of LH patients vs. LH controls resulted in no predictors passing initial criterion for inclusion, indicating that patients and controls cannot be differentiated on LH CxT. A model with a strong association between the predictors and RH patient vs. RH control groups, *r*^*2*^=0.8, included the CxT of the caudal middle frontal, frontal pole, lateral occipital, lingual, pars orbitalis, and transverse temporal regions. A model with *r*^*2*^=0.89 which included CxT of entorhinal, rostral middle frontal, and superior temporal regions differentiated LH from RH patients. No viable model was able to separate the patients into ILAE high vs. low binned scores.

### Cortical Surface Area

**Figure 3:**
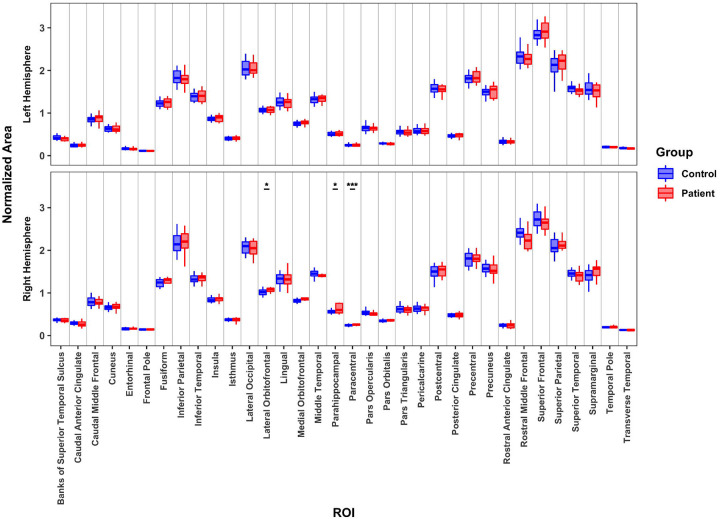
Median normalised cortical surface area for 34 cortical regions for patients with a preserved left hemisphere or right hemisphere relative to their matched controls. *: *p*<0.05; ***: *p*<0.001.

As depicted in Figure 3, compared with their matched controls, patients with a preserved LH had no differences in CSA, whereas patients with a preserved RH had larger CSA in lateral orbitofrontal, paracentral, and parahippocampal cortices. The two patient groups differed from one another with the preserved LH patients having greater CSA in three regions (pars opercularis, rostral anterior cingulate and transverse temporal) and the preserved RH patients having greater CSA in four regions (frontal pole, inferior parietal, parahippocampal and pars orbitalis). (See Supplementary Table 3 for individual *p* and *BF* values.)

In a forward logistic regression analysis with CSA of all 34 areas entered as independent measures for the LH patients and matched controls, no model could be derived that associated predictor (region) with outcome (group membership). For the RH patients and controls, the most predictive model contained a high *r*^*2*^=0.83 and included the CSA of the entorhinal, lateral orbitofrontal, paracentral, rostral middle frontal, and superior temporal regions. The two patient groups were differentiable by a model containing just the CSA of the pars orbitalis area, with *r*^*2*^=0.95, and a model with *r*^*2*^=0.33 including just the paracentral area predicted ILAE outcome score.

### Cortical Volume

**Figure 4:**
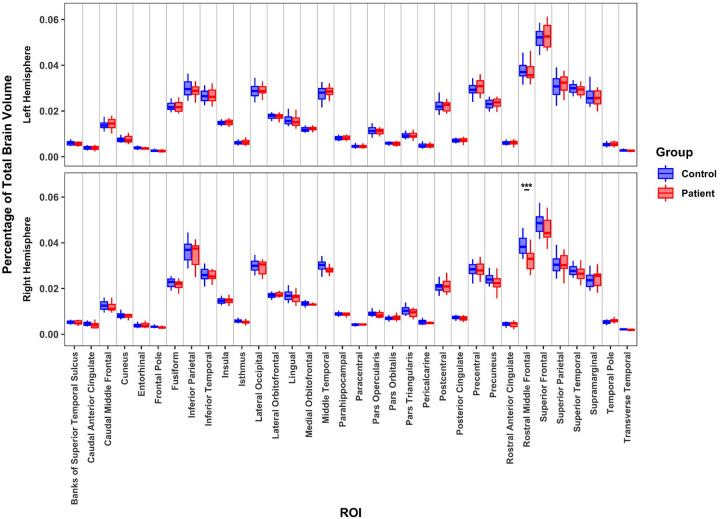
Median region volume (normalised by total volume) for each of 34 cortical regions for patients with a preserved left hemisphere or right hemisphere relative to their matched controls. ***: *p*<0.001.

On the final dependent measure, CV, as shown in Figure 4, the GLM revealed no differences between the LH patients and the LH of the controls. A difference in rostral middle frontal volume was observed between the RH patients and the RH of the controls. A direct comparison between the two patient groups indicated four regions with less volume in the RH than the LH patient group (inferior parietal, pars opercularis, transverse temporal, rostral anterior cingulate) and greater volume in the RH than LH patient group in two regions (pars orbitalis, superior frontal). (See Supplementary Table 4 for individual *p* and *BF* values.)

A forward logistic regression analysis with the measures of CV for each of the 34 regions entered predicting the differences between LH patients and controls had no significant predictors. A model with *r*^2^=0.57, which included the rostral middle frontal and pars orbitalis volume predicted group membership for the RH patients vs. RH controls. The RH and LH patients were well differentiated by a model containing pars opercularis and pars orbitalis with *r*^2^=0.9. ILAE score was also highly predicted by a model containing CV of paracentral, pars triangularis, and superior parietal volume, with *r*^2^=0.71.

**Figure 5:**
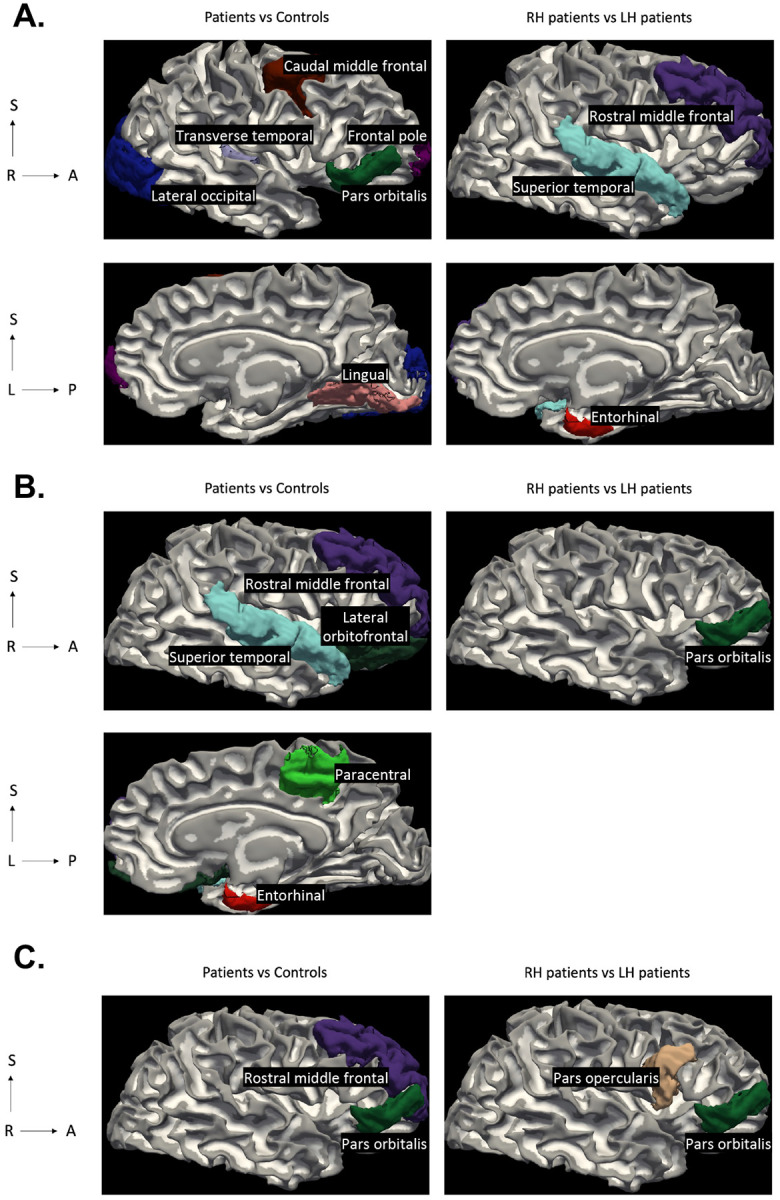
Regions that significantly distinguished between groups (patients versus controls in left columns, right hemisphere patients versus left hemisphere patients in right columns) per binary logistic regression modelling for (A) cortical thickness, (B) cortical surface area, and (C) cortical volume.

For a summary of all logistic regression analyses, see Figure 5.

### Subcortical Regional Morphometrics

We used the FreeSurfer atlas to segment the preserved hemisphere in patients and both hemispheres in controls, into nine subcortical regions, namely, the nucleus accumbens (here referred to as accumbens), amygdala, caudate, cerebellum, hippocampus, pallidum, putamen, thalamus, and ventral diencephalon; and extracted the volume of these regions.

**Figure 6:**
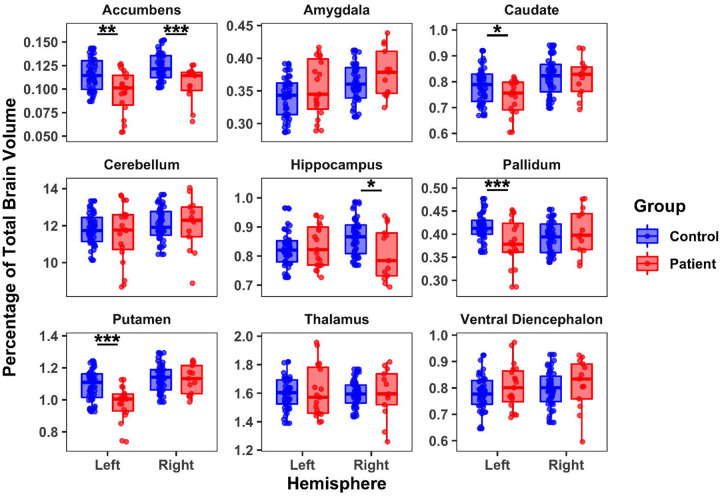
Subcortical regional volumes (normalised) for patients with a preserved left hemisphere or right hemisphere relative to their matched controls *: *p* < .05; **: *p* < .01; ***: *p* < .001.

Compared with the LH of controls, patients with a preserved LH showed a significant volume reduction of the accumbens, caudate, pallidum, and putamen. Patients with a preserved RH had significantly reduced volume in the accumbens and hippocampus compared to controls. The LH patient group had significantly lower volume in the caudate and putamen relative to the RH patient group (Figure 6). (See Supplementary Table 5 for individual *p* and *BF* values.)

To evaluate which of these nine subcortical measures, singly or in combination, best predicted group membership, we used the same forward binary logistic regression procedure as above. The model differentiating LH patients and LH controls yielded a model which included putamen and accumbens, with *r*^*2*^=0.44, revealing a strong relationship between these two predictors and group membership. The same analysis for RH patients and RH controls included only the accumbens and yielded a *r*^*2*^=0.28 indicating a moderate relationship between this predictor and outcome. A regression model that included the putamen volume alone differentiated strongly between the preserved RH vs. LH patients, with *r*^*2*^=0.52. Last, no model including volume of the subcortical measures was reliable in classifying patients into the two ILAE (high, low) bins.

In sum, the GLM results clearly differentiated the LH and RH patients from their matched controls on four and two of the nine subcortical regions, respectively, with the accumbens having lower volume for both patient groups vs. controls. The preserved LH patients had smaller volume than the RH patients in the putamen and caudate. The regression model identified the accumbens and the putamen as predicting LH patients from controls, and the accumbens alone as predictive of group membership in RH patients vs. controls. The putamen alone separated the two patient groups, which is unsurprising given the two direct patient-controls comparisons, and the ILAE outcome could not be predicted by any variable/s. These findings indicate that the accumbens and/or putamen are the key subcortical structures (with caudate and pallidum for LH patients) that differentiate patients from controls, and the putamen is key for differentiating side of preserved hemisphere in the patients.

### Analysis Excluding Ablation Cases

One possible explanation for the differences between the findings for the LH and RH patients is that the number of cases of resection and ablation vary for the two groups, with a larger proportion of the LH than RH patients having an ablation rather than a resection: LH – 7 ablation, 12 resection; RH – 2 ablation, 11 resection. Considering this, we recomputed all the binary logistic regression analyses with just the resection cases (12 LH, 11 RH). Notwithstanding the loss of statistical power with this data reduction, the findings using the three gross measures (LV, GM, and WM) largely mirrored that of the analysis including all patients: whereas no model could predict group membership between LH patients and controls or between LH patients and RH patients, a model with LV and GM with *r*^2^ = 0.48 discriminated between the RH patients and RH controls.

Additionally, the analysis of the cortical regions with just the resection patients revealed largely similar results to that conducted with all the patients. With respect to CxT, a relatively weak model of *r*^2^=0.27–which included the fusiform and the inferior parietal regions–predicted group membership for LH patients vs. LH controls. A stronger association between group membership (patient vs. control) for the RH was observed with the CxT of just two areas, the frontal pole and posterior cingulate playing a predictive role, and a strong prediction of side of preserved hemisphere (LH vs. RH) was obtained for separating the two patient groups. For CSA, no model predicted patients vs. controls for the LH comparisons. However, a model with *r*^2^=0.76 (lateral orbitofrontal, parahippocampal, rostral middle frontal, superior temporal) separated patients and controls in the RH comparisons, and a very strong model of *r*^2^=0.9 just with the pars orbitalis separated the two patients groups. Last, the findings of differences in CV were well substantiated in the no-ablation analysis.

Last, the regression analysis using the subcortical regions essentially replicated the analysis with all the patients and perhaps yielded results that were even statistically stronger. A model with *r*^2^=0.56 including the accumbens and putamen separated the LH patients from their controls; a model with *r*^2^=0.33 and including the accumbens separated the RH patients from their controls, while a model with *r*^2^=0.66 and including only the putamen separated the two patient groups.

### Correlations Between Gross Morphometrics and Cortical/Subcortical Morphometrics

In this final analysis, we examine whether changes in gross measures, for example, the size of the LVs are statistically associated with subcortical or cortical measures. Because the cranium is a fixed size cavity, a large increase in one major feature/tissue like CSF and LV volume will necessarily induce a large decrease in the rest, such neighbouring white matter. To examine this, we performed bivariate correlations (with multiple comparison correction *p*<.0002) between the gross measures, the volumes of subcortical regions, and the three dependent measures for each of the 34 cortical parcels, and we did this separately for all patients (*N*=32), and then only LH patients and only RH patients. In no analysis did any correlation survive the most stringent multiple comparison.

## Discussion

We undertook a comprehensive characterisation of the structural integrity of the post-surgical preserved hemisphere in a sample of DRE paediatric patients. Studies of postsurgical brain anatomy in DRE adults have yielded conflicting findings: whereas some have reported reduction, and even reversal, of progressive cortical thinning (i.e. an increase in CxT)^5, 6, 10, 13,48^ and cortical atrophy,^5^ others have noted ongoing, progressive atrophy,^13^ even in regions remote from the resection site or hemisphere^7, 12^ (although concerns regarding the methodologies have been expressed).^7^ Almost all such studies have been carried out in adults with temporal lobe epilepsy or anterior temporal lobectomy, and have largely focused on just one variable, such as CxT or CV (but see Zhao et al.^15^ for both).

Here, we elucidated the morphometry of the preserved hemisphere in individuals following surgery for the management of epileptic seizures in childhood, an age of greater opportunity for plasticity than in adulthood. A clear depiction of the non-resected hemisphere is increasingly pressing as surgery for those with DRE, especially during childhood, is becoming a first-line form of intervention.^16–18^

We recruited 32 post-surgical patients, with different forms of intervention ranging from large resections such as hemispherectomy to more focal alterations such as after robot-assisted laser ablation and compared their preserved hemisphere to that of matched controls. We then compared the morphometry of the patients with preserved LH with that of patients with preserved RH. We derived a host of morphometric measures from gross indices of LV, GM, and WM volumes, multiple indices of 34 cortical parcels, and volumes of nine subcortical regions. We also examined differences as a function of which hemisphere was preserved, redid the analysis to exclude laser ablation (more focal intervention), and examined correlations across the various metrics. Statistical analyses were conducted using both GLM and binary logistic regression analyses to provide different vantage points on the data, and the results were largely, although not always perfectly, converging.

Several key findings emerged from the data (see summary in Table 2).

First, both LH and RH patient groups differed from their matched controls on multiple gross anatomical measures with larger LVs and reduced WM (and GM in RH patients). The volume of GM alone successfully predicted whether the LH vs. RH was preserved with less atrophy in the LH than RH.

For the analysis of the 34 cortical regions, we adopted multiple measures for quantification (CxT in mm, CSA in mm^2^ and CV in mm^3^).

Patients with a preserved LH were more like their controls on all three dependent measures, and the only statistic that differentiated the patients from the controls (in binary regression but not GLM) was the CSA of the superior temporal region. The cortex of patients with a preserved RH differed from their controls on all three dependent measures in both binary logistic and GLM analyses. For example, measures of cortical thickness revealed reductions in a host of frontal, temporal, and occipital areas and in the isthmus, insula, and precuneus. Counterintuitively, the patients had larger CSA than controls in lateral orbitofrontal, parahippocampal, and paracentral areas, and a subset of these areas predicted the group membership (patient vs. control) in the best fit regression model. Lastly, the RH patients evinced reductions in the CV of the rostral middle frontal area (and no obvious parietal region differences), which, together with the pars orbitalis gave rise to good assignment of membership into patients vs. controls.

A direct comparison between the two patient groups revealed a host of differences with greater reduction in CxT in frontal, entorhinal, and superior temporal regions, along with, for preserved RH vs .LH, reduced CSA in frontal cingulate and transverse temporal regions, but also greater CSA in, for example, frontal pole, parahippocampal and pars orbitalis. Last, those with a preserved RH had smaller CV (more atrophy) in a set of frontal and temporal regions, and, consistent with the CSA measurements, larger volume in the inferior parietal and pars orbitalis regions. It is particularly noteworthy that between patient groups, differences in the pars orbitalis and the pars opercularis manifested as key sites in the CSA and CV GLM and regressions analyses.

With respect to the nine subcortical regions, the volume of the putamen and accumbens (along with other structures such as pallidum, caudate, and hippocampus) was reduced in both patient groups vs. controls, and the volume of the putamen alone (less volume in LH than RH) was able to differentiate the two patient groups from each other.

Last, there were no correlations between gross measures and any other subcortical or cortical metrics that survived stringent familywise correction indicating independence between the morphometric changes of LV, WM and GM volume, and other measurements.

### Right hemisphere versus left hemisphere differences

The key findings include the presence of numerous differences in all three dependent measures between those with a preserved RH and their matched controls or between them and those with a preserved LH. In almost all instances, the preserved RH has reductions in CxT and CV but, surprisingly, has larger CSA in a few regions including orbitofrontal and parahippocampal regions. This relative decrement in the preserved RH is accompanied by some alterations in subcortical regions, namely in the accumbens, caudate, and putamen.

Because the two groups were matched on age, gender, age at first surgery, age of seizure-onset age, and ILAE scores, and age and gender were included as covariates in all GLM analyses, none of these factors obviously account for the hemispheric differences. One potential difference is that the data were acquired on two different scanners, with arguably different resolution, but this, too, does not obviously explain the asymmetry as both patient groups were roughly equally represented on each scanner (Siemens Verio: 4 RH, 9 LH and Siemens Prisma: 8 RH, 8 LH) and, moreover, the data were harmonised across magnets.

As discussed above, another obvious possibility concerns a possible difference in the size of the resection in each group, with more cases with small lesions (thermal ablation) *N*=7 and *N*=2 in the LH and RH patient groups, respectively. The re-analysis of the data (see Results), with only the frank resection and no ablation patients, rules out this potential confound. In fact, despite the reduction in statistical power by removing the ablation patients, we replicate almost all the results of the analyses with the data including all patients. The asymmetry is also not obviously explained by differences in lobar resections: both LH and RH had four patients with temporal resections and two patients with frontal lobe resections. The LH and RH groups had one and two patients with resections involving occipital cortex, and two and one patients involving parietal cortex, respectively (the number of cases exceed 32 as patients often have multi-lobar resections), and again, the patient groups are relatively well-matched and the lobar involvement does not explain the hemispheric asymmetry.

### Right hemisphere versus left hemisphere cortical regional differences

Scrutiny of the specific regional differences between those with preserved LH vs. RH may offer a possible explanation of the hemispheric asymmetry. The primary regional differences arise in the pars orbitalis and pars triangularis, but also in the caudal middle and superior frontal, middle and superior temporal, and transverse temporal regions. These findings, derived from an unbiased and whole brain data-driven analysis appear to converge largely on regions that are implicated in language function. Resecting some, most, or all of the LH (preserved RH group), and, likely, the native dominant language areas in childhood, is likely the source of alterations of LH homologues of language areas in the preserved RH. In contrast, a resection of some, most, or all of the RH (preserved LH group) results in remarkably few structural or anatomical differences relative to the matched controls’ LH and, indeed, such children with a preserved LH have been shown to have better cognitive and language outcomes.^49^

The hypothetical account, then, is that after LH cortical resection, regions of the preserved RH that are homotopic with LH language regions come to assume language functions. The process of accommodating language function in the RH (which likely has some nascent or “shadow” of language function^50, 51^) potentially trigger the morphometric changes in the RH. Much evidence attests to the fact that, in early childhood, both hemispheres are predisposed to language function. For example, in significant contrast to the marked aphasias evident in adults with brain injury, children with unilateral brain injury performed within the normal range of language function, and this was true independent of hemisphere of injury.^52^ Likewise, adolescents and young adults who suffered a perinatal ischemic stroke to the LH nevertheless displayed sentence processing abilities equal to that of non-neurological controls, presumably a result of RH language engagement.^51, 53^ The idea of RH plasticity for language has also been confirmed in neuroimaging studies: individuals with peri- or prenatal periventricular damage to the LH evinced RH blood-oxygen-level-dependent activation during a silent word generation task, and the extent of the activation equaled that of the LH in right-handed controls.^54^ Findings such as these attest to the remarkable plasticity and potential for functional reorganisation of language to the RH homotopic frontotemporal regions.

What remains to be explained is why we see reduction in these RH homotopic language regions rather than maintenance or even expansion, which is often associated with the assumption of a new function. Also, the changes in the RH are widespread: almost 50% of RH cortical regions show significant reduction in CxT. Of note, almost all of these areas in the RH are homotopic with or proximal to standard LH language areas (for example, lateral orbitofrontal, middle temporal, rostral middle frontal, superior frontal, and transverse temporal areas). Presumably, as is also true over the course of normal development, cortical regions are pruned as functions are acquired through the removal of inefficient synapses, dendrites, and neurons. Many have suggested that the cortical tissue loss reflects improved neural processing by optimising brain circuits for particular operations.^55^ Cortical thinning is coupled with morphological changes,^55^ and, in childhood, GM thinning,^56^ specifically, is associated with changes in behaviour, which may be associated with increases in WM.^57^ In a longitudinal study, changes of CxT asymmetry in the triangular part of the inferior frontal gyrus, constituting a portion of Broca’s area in the LH, is suggestive of a neural correlate of language improvement in children’s brains at roughly ages 5–7 years.^58^ Changes to areas that are proximal to RH homotopic language areas may reflect the imprecision of the changes (for example, in pruning of neurons and associated dendrites).

Given the cross-sectional, rather than longitudinal design, of the current study, we are unable to evaluate changes over time and their relationship to behaviour. Reversal of progressive thinning and recovery from atrophy and thinning^15^ and specifically, in post-surgical vs. presurgical cortical regions, could not be assessed. Because of our one-time snapshot, whether the profiles of the childhood DRE brains improved specifically over presurgical profiles or not and the relationship between these changes over time, hemisphere of resection, and cognitive (specifically language) outcome remain unaddressed and serve as rich fodder for future studies.

## Supplementary Material

Supplement 1

## Figures and Tables

**Table 1:** Patient information

Age (yr)	Gender	Age at surgery (yr)	Age of seizure onset (yr)	ILAE Scale (Binned)	Surgery
**Left Surgery Patients/Preserved Right**					
11.5	Male	1	0	Low	Hemispherectomy
12.5	Male	0	-	Low	Evacuation of temporal hematoma
13.9	Female	13	6	Low	Occipital and parietal lobectomy
14.7	Male	13	12	Low	Temporal lobectomy with preservation of medial structures & gross total resection of enhancing medial temporal lobe tumor
15.1	Female	14	8	Low	Frontal lobe resection
15.6	Female	13	6	Low	Functional hemispherectomy
15.7	Male	15	7	Low	Frontal lesionectomy with corticectomy
16.1	Male	10	0	High	Temporal lobectomy with amygdalohippocampectomy
16.7	Female	16	13	High	Robot-assisted stereotactic laser ablation of mesial temporal lobe including amygdala and hippocampus (x2)
16.8	Male	4	0	Low	Functional hemispherectomy
19.8	Female	4	1	Low	Hemidecortication
20.5	Male	18	13	Low	Resection of T1 gyrus & Heschl gyrus seizure onset zone
22.2	Female	17	11	Low	Amygdalohippocampectomy, anterior temporal lobectomy, & resection of mesial temporal lobe tumor
**Right Surgery Patients/Preserved Left**					
7.8	Female	3	2	Low	Hemispherotomy
7.8	Male	6	4	Low	Occipital lobectomy, posterior temporal lobectomy, & resection of medial inferior temporal lobe tumor
10.9	Female	10	4	High	Robot-assisted stereotactic laser thermal ablation of frontal seizure onset zone
11.3	Male	10	6	High	Robot-assisted stereotactic laser thermal ablation of amygdala & hippocampus
11.6	Female	4	4	High	Temporal lobe lobectomy with amygdalohippocampectomy
13.1	Female	12	10	High	Robot-assisted stereotactic laser thermal ablation of operculum
14.4	Female	13	3	High	Robot-assisted stereotactic laser thermal ablation of amygdalar seizure onset zone
14.8	Female	8	0	Low	Functional hemispherectomy
15.0	Female	10	9	Low	Resection of frontal operculum, frontal pole, & inferior frontal lobe
15.4	Male	15	4	High	Robot-assisted stereotactic laser thermal ablation of mesial temporal lesion
16.1	Female	1	0	Low	Hemispherotomy
16.6	Male	16	11	Low	Robot-assisted stereotactic laser ablation of occipito-temporal seizure onset zone (x2)
17.0	Female	15	13	Low	Anterior temporal lobectomy & hippocampectomy
17.4	Female	15	10	Low	Resection of parietal lobe, premotor & supplementary motor area
17.8	Male	17	16	Low	Gross total resection of frontal brain tumor
18.3	Male	12	7	Low	Parietal lobectomy & posterior temporal lobectomy
18.7	Female	9	0	Low	Hemispherectomy
19.4	Male	14	6	High	Partial resection of parietooccipital tumor (x2)
20.1	Male	18	10	Low	Robot-assisted stereotactic laser thermal ablation of insulo opercular cortex

Note: Classes 1–3 are binned into the “Low” ILAE scale (better outcome) and Classes 4–6 are binned into the “High” scale (poorer outcome).

**Table 2:** Summary of all results for univariate and multivariate analyses

Measures	LH patients/LH controls	RH patients/RH controls	RH patients/LH patients
**Gross volumes**			
**General linear model**			
Lateral Ventricle	patients > controls	patients > controls	n.s.
Grey Matter	n.s.	patients < controls	RH < LH
White Matter	patients < controls	patients < controls	n.s.
**Logistic regression**	LV and WM	GM and LV	GM
**Cortical**			
**Thickness**			
**General linear model**	n.s.	patients < controls: caudal middle frontal, cuneus, frontal pole, fusiform, insula, isthmus, lateral orbitofrontal, lingual, middle temporal, paracentral, posterior cingulate, precuneus, rostral middle frontal, superior frontal, transverse temporal	RH < LH: caudal middle frontal, rostral middle frontal, superior frontal
**Logistic regression**	n.s.	caudal middle frontal, frontal pole, lateral occipital, lingual, pars orbitalis, transverse temporal	entorhinal, rostral middle frontal, superior temporal
**Surface area**			
**General linear model**	n.s.	patients > controls: lateral orbitofrontal, parahippocampal, paracentral	LH > RH: pars opercularis, rostral anterior cingulate, transverse temporal;
			RH > LH: frontal pole, inferior parietal, parahippocampal, pars orbitalis
**Logistic regression**	superior temporal	entorhinal, lateral orbitofrontal, paracentral, rostral middle frontal, superior temporal	pars orbitalis
**Volume**			
**General linear model**	n.s.	patients < controls: rostral middle frontal area	LH > RH: pars opercularis, rostral anterior cingulate, superior frontal, transverse temporal;
			RH > LH: inferior parietal, pars orbitalis
**Logistic regression**	n.s.	rostral middle frontal, pars orbitalis	pars opercularis, pars orbitalis areas
**Subcortical volumes**			
**General linear model**	patients < controls; accumbens, caudate, pallidum, putamen	patients < controls: accumbens, hippocampus	RH > LH: caudate, putamen
**Logistic regression**	accumbens, putamen	accumbens	putamen

n.s.: not significant

LH: left hemisphere

RH: right hemisphere

## Data Availability

A DOI has been reserved on Carnegie Mellon University’s KiltHub repository (operated via Figshare): 10.1184/R1/24153423. All relevant data and code will be published in this repository upon publication of the article.
