# Morphometrics of the preserved post-surgical hemisphere in pediatric drug-resistant epilepsy

**DOI:** 10.1101/2023.09.24.559189

**Published:** 2023-12-15

**Authors:** Michael C. Granovetter, Anne Margarette S. Maallo, Christina Patterson, Daniel Glen, Marlene Behrmann

**Affiliations:** 1 Department of Psychology and Neuroscience Institute, Carnegie Mellon University, Pittsburgh, PA, USA 15213; 2 School of Medicine, University of Pittsburgh, Pittsburgh, PA, USA 15213; 3 Department of Pediatrics, University of Pittsburgh, Pittsburgh, PA, USA 15213; 4 Scientific and Statistical Computing Core, National Institute of Mental Health, Bethesda, MD, USA 20892; 5 Department of Ophthalmology, University of Pittsburgh, Pittsburgh, PA, USA 15213

## Abstract

**Importance:**

Structural integrity of cortex following cortical resection for epilepsy management has been previously characterized, but only in adult patients.

**Objective:**

This study sought to determine whether morphometrics of the preserved hemisphere in pediatric cortical resection patients differ from non-neurological controls.

**Design:**

This was a case-control study, from 2013-2022.

**Setting:**

This was a single-site study.

**Participants:**

32 patients with childhood epilepsy surgery and 51 age- and gender-matched controls participated.

**Main Measure(s):**

We quantified morphometrics of the preserved hemisphere at the level of gross anatomy (lateral ventricle size, volume of gray and white matter). Additionally, cortical thickness, volume, and surface area were measured for 34 cortical regions segmented with the Desikan-Killiany atlas, and, last, volumes of nine subcortical regions were also quantified.

**Results:**

13 patients with left hemisphere (LH) surgery and a preserved right hemisphere (RH) (median age/median absolute deviation of age: 15.7/1.7 yr; 6 females, 7 males) and 19 patients with RH surgery and a preserved LH (15.4/3.7 yr; 11 females, 8 males) were compared to 51 controls (14.8/4.9 yr; 24 females, 27 males). Patient groups had larger ventricles and reduced total white matter volume relative to controls, and only patients with a preserved RH, but not patients with a preserved LH, had reduced total gray matter volume relative to controls. Furthermore, patients with a preserved RH had lower cortical thickness and volume and greater surface area of several cortical regions, relative to controls. Patients with a preserved LH had no differences in thickness, volume, or area, of any of the 34 cortical regions, relative to controls. Moreover, both LH and RH patients showed reduced volumes in select subcortical structures, relative to controls.

**Conclusions and Relevance:**

That left-sided, but not right-sided, resection is associated with more pronounced reduction in cortical thickness and volume and increased cortical surface area relative to typically developing, age-matched controls suggests that the preserved RH undergoes structural plasticity to an extent not observed in cases of right-sided pediatric resection. Future work probing the association of the current findings with neuropsychological outcomes will be necessary to understand the implications of these structural findings for clinical practice.

## Introduction

Surgical resection effectively reduces seizures in drug-resistant epilepsy (DRE).^1^ Whether there are postoperative changes in the structural integrity of non-resected cortex is unclear. Post-surgically, reduction in cortical thickness (CxT), a proxy for structural integrity,^2^ has been identified beyond the epileptogenic zone/lobe, even in the preserved hemisphere. In contrast, in patients with temporal lobe epilepsy, pre-surgical CxT reduction can be reversed to within normal limits, and the extent of reversal is correlated with better seizure outcomes.^3-8^ Additionally, reversed cortical volume (CV) loss has been observed postsurgically, albeit only in patients with anterior temporal lobectomy,^9^ but postsurgical deceleration of cortical atrophy has also been reported.^3, 5, 10, 11^ The discrepant postoperative atrophy versus recovery warrants further study. If the surgical outcome is of progressive—rather than reduced—atrophy, alternative or additional disease-modifying treatments might be needed to halt progression.

Here, we compared postsurgical morphometry of the preserved hemisphere of children with DRE and non-neurological controls on (i) gross volumes of the lateral ventricles (LV), gray matter (GM), and white matter (WM); (ii) CxT, CV, and cortical surface area (CSA) of 34 regions; and (iii) volume of nine subcortical structures. CxT, CV, and CSA are commonly utilized in studying DRE^12^ and have distinct genetic profiles and lifespan trajectories.^13-15^ CxT linearly decreases with age;^16^ CSA and CV follow a curvilinear trajectory with CSA peaking before CV.^14^ Complementary information gleaned from these measures can offer insights into structural perturbations. Lastly, although postsurgical reduction in cortical thinning affects both hemispheres equally in adults,^3^ here, we also determined whether, in children, postsurgical structural differences depended on which hemisphere is preserved. We focused analyses on the preserved hemisphere, as ipsilesional cortex may have structural abnormalities from surgery itself, confounding postoperative structural plasticity that potentially occurs post-resection.

## Materials and methods

### Participants

Thirty-two pediatric cortical resection or ablation patients participated: 13 with LH surgery and a preserved RH (median age/median absolute deviation of age: 15.7/1.7 yr; 6 females, 7 males) and 19 with RH surgery and a preserved LH (median age/median absolute deviation of age: 15.4/3.7 yr; 11 females, 8 males; see eTable 1 for details). “RH patients” are those with left-sided resections but a preserved RH, and likewise “LH patients” are those with a preserved LH, with the labels indicating the hemisphere from which the measurement is taken.

Patient groups were matched on age (*F*(1,30)=1.12; *p*=.30), gender (*z*(30)=0.65; *p*=.51), initial surgery age (*F*(1,30)=0.03; *p*=.87), seizure-onset age (*F*(1,29)=0.01; *p*=0.93), and binned International League Against Epilepsy (ILAE) outcome scales^17^ (*z*(30)=1.29; *p*=.20). Patients were recruited from Children’s Hospital of Pittsburgh or via the Pediatric Epilepsy Surgery Alliance. Patients were matched with 51 control participants (median age/median deviation of age: 14.8/4.9 yr; 24 females, 27 males) on age (*F*(1,81)=1.18; *p*=.28) and gender (*z*(81)=0.54; *p*=.59).

Carnegie Mellon University and University of Pittsburgh Institutional Review Boards approved the study.

### Protocol

T1-weighted images were acquired using a magnetization-prepared rapid acquisition gradient echo sequence (1-mm isotropic resolution, TE=1.97 ms, TR=2300 ms, scan time≅5 min) with either a 3T Siemens Verio scanner (32-channel head coil; 36 controls, 13 patients) or Siemens Prisma 3T scanner (64-channel head coil; 15 controls, 19 patients).

### Outcome Measures

Images were motion-corrected, normalized, and segmented with FreeSurfer (v7.1.0)^18-20^ before manual inspection. As FreeSurfer often does not accurately segment hemispherectomy/hemispherotomy/hemidecortication brains, for these cases, the intact hemisphere was mirrored using affine/non-linear transformations robust to aberrations, and only the preserved hemisphere was analysed.^21, 22^

Morphometric measures from the preserved hemisphere of patients and each hemisphere of controls were as follows: (i) total GM, WM, and LV volumes; (ii) cortical morphometry for 34 regions, parcellated according to the Desikan-Killiany atlas;^23, 24^ and (iii) nine subcortical structures’ volumes.^25, 26^ We did not normalize CxT (as in Westman et al.^27^), but did so for CSA by dividing by the mean CSA of all of regions in that hemisphere, and for CV as the percentage of total hemisphere volume (sum of GM, WM, and LV volumes).^28-30^

### Analysis

Data were analyzed in R^31^ (for packages, see eTable 2) and SPSS 29.0.1.0. To harmonize data across the two scanners, data were modelled as a linear combination of group, age, gender, and scanner, assuming scanner effects have both additive and multiplicative factors.^32, 33^ For each measure and ROI, data were winsorized: values above the 95th and below the 5th percentile of the distribution were replaced with approximate corresponding percentiles (separately for controls and patients, and by hemisphere).

Given the small sample sizes from this relatively rare patient population, for each comparison, permutation testing was implemented by randomly shuffling the group label, and a general linear model (GLM) was fit with group as the primary predictor and age and gender as covariates. This was repeated 1,000 times to create a distribution of β-coefficients for the group effect. *p*-value was the percentage of occurrences in which the absolute value of the β-coefficient from the simulated distribution exceeded the absolute value of the true β-coefficient (α=.05). Per measure, Benjamini-Hochberg correction^34^ was applied to *p*-values across ROIs. Bayes factor (BF) was also computed by comparing the model with the group term to a null model without the group term.^35, 36^

To elucidate which measures, alone or in combination, predicted group membership for each patient group and the matched hemisphere of the controls and between LH and RH patient groups, we conducted forward binary logistic regression analyses with *p*<.05 for entry criterion. We also predicted ILAE outcome (high versus low) for the patients using this multivariate approach. *r*^2^ of models are provided using the Nagelkerke measure, and no other factors are entered when the model change was less than .001.

## Results

### Gross Morphometrics

**Figure 1:**
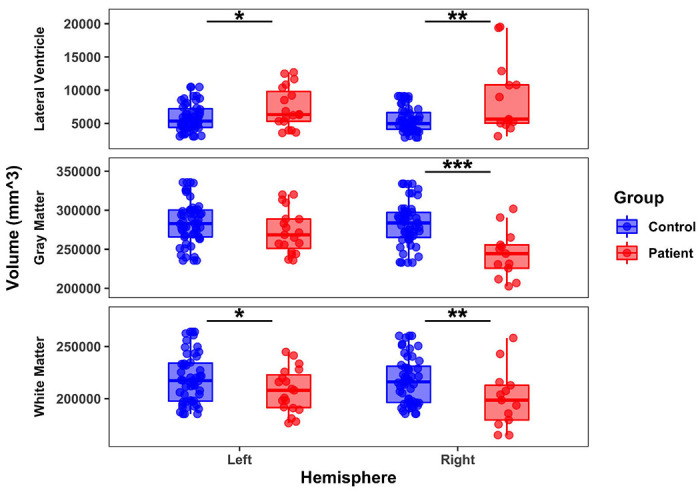
Volume of (top) lateral ventricles, (middle) grey matter, and (bottom) white matter for patients and controls, separately for the preserved left and right hemispheres. *: *p*<.05; **: *p*<.01; ***: *p*<.001. Each dot represents a single participant, and the solid horizontal line in the box indicates the median of the group.

Relative to controls’ LH, patients’ LH had larger LV volume (*p*=.02, *BF*=0.57), smaller WM volume (*p*=.05, *BF*=1.02), and no GM volume difference (*p*=.27, *BF*=4.37). Relative to controls’ RH, patients’ RH had larger LV volume (*p*<.01, *BF*=0.01), smaller WM volume (*p*<.01, *BF*=5.56*10^−2^), and smaller GM volume (*p*<0.001, *BF*=1.6*10^−3^). LH and RH patients did not differ on LV volume (*p*=.55, *BF*=4.54) or WM volume (*p*=.13, *BF*=1.39). However, RH patients had smaller GM volume than LH patients (*p*<.01, *BF*=1.98*10^−2^). See Figure 1 for data and eTable 3 for statistics.

Evaluating whether LV, GM, and WM, singly or in combination predicted group membership, LH patients and LH controls were undifferentiable. RH patients and RH controls’ group membership could be predicted by GM and LV (*r*^2^=0.5), in that order, but not by WM. GM alone best predicted hemisphere preserved when comparing the two patient groups (*r*^2^=0.28). No predictor/s differentiated patients’ ILAE outcome scales.

### Cortical Morphometrics

#### Thickness

**Figure 2:**
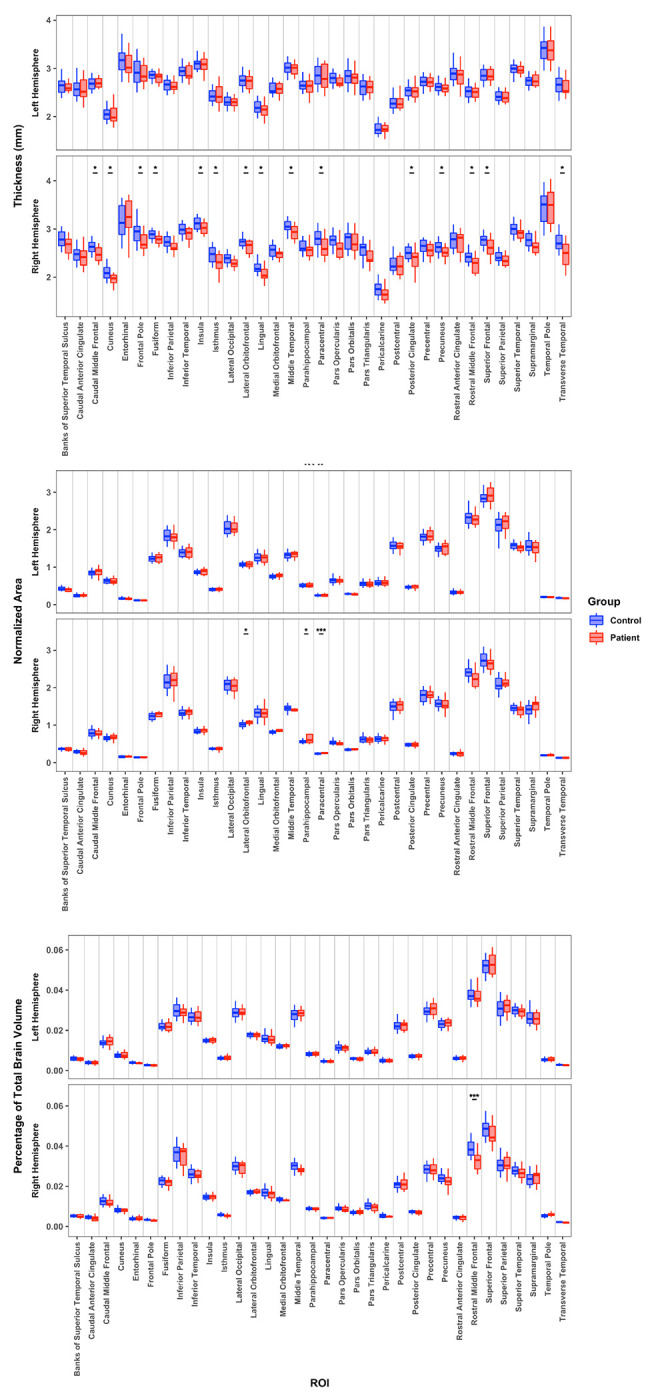
Median cortical thickness (top), normalized cortical surface area (middle), and region volume (normalized by total volume; bottom) for 34 cortical regions for patients with a preserved left hemisphere or right hemisphere relative to their matched controls. *: *p*<0.05; **: *p*<0.01; ***: *p*<0.001.

Relative to controls’ LH, patients’ LH showed no differences in CxT in any region whereas, compared to controls’ RH, patients’ RH had reduced CxT in 15 of the 34 regions and no region with greater thickness. Relative to LH patients, RH patients had reduced CxT in three regions: the caudal middle frontal, rostral middle frontal, and superior frontal regions. See Figure 2 for data and eTables 4-5 for statistics.

Forward logistic regression analysis with CxT indices of all 34 regions could not differentiate LH patients vs. LH controls. However, a model with CxT of caudal middle frontal, frontal pole, lateral occipital, lingual, pars orbitalis, and transverse temporal regions differentiated RH patients vs. RH controls (*r*^2^=0.8). A model with CxT of entorhinal, rostral middle frontal, and superior temporal regions differentiated LH from RH patients (*r*^2^=0.89). No predictor/s differentiated patients’ ILAE outcome scales.

#### Surface Area

Compared with their matched controls, LH patients had no differences in CSA, whereas RH patients had greater CSA in lateral orbitofrontal, paracentral, and parahippocampal cortices. The patient groups also differed from one another: LH patients had greater CSA in the pars opercularis, rostral anterior cingulate, and transverse temporal regions while RH patients had greater CSA in the frontal pole, inferior parietal, parahippocampal, and pars orbitalis regions. See Figure 2 for data and eTables 6-7 for statistics.

Forward logistic regression analysis with CSA indices of all 34 regions could not differentiate LH patients vs. LH controls. However, a model with CSA of entorhinal, lateral orbitofrontal, paracentral, rostral middle frontal, and superior temporal regions differentiated RH patients vs. RH controls (*r*^2^=0.83). A model with CSA of pars orbitalis differentiated LH from RH patients (*r*^2^=0.95), and a model with CSA of paracentral cortex predicted ILAE outcome score (*r*^2^=0.33).

#### Volume

Compared with matched controls, LH patients had no differences in CV, whereas RH patients had greater CV in rostral middle frontal cortex. The two patient groups differed from one another: LH patients had greater CV in the inferior parietal, pars opercularis, transverse temporal, and rostral anterior cingulate regions while RH patients had greater CV in the pars orbitalis and superior frontal regions. See Figure 2 for data and eTables 8-9 for statistics.)

Forward logistic regression analysis with CV indices of all 34 regions could not differentiate LH patients vs. LH controls. However, a model with CV of rostral middle frontal and pars orbitalis regions differentiated RH patients vs. RH controls (*r*^2^=0.57). A model with CV of pars opercularis and pars orbitalis differentiated LH from RH patients (*r*^2^=0.9), and a model with CV of the paracentral, pars triangularis, and superior parietal regions predicted ILAE outcome score (*r*^2^=0.71).

**Figure 3:**
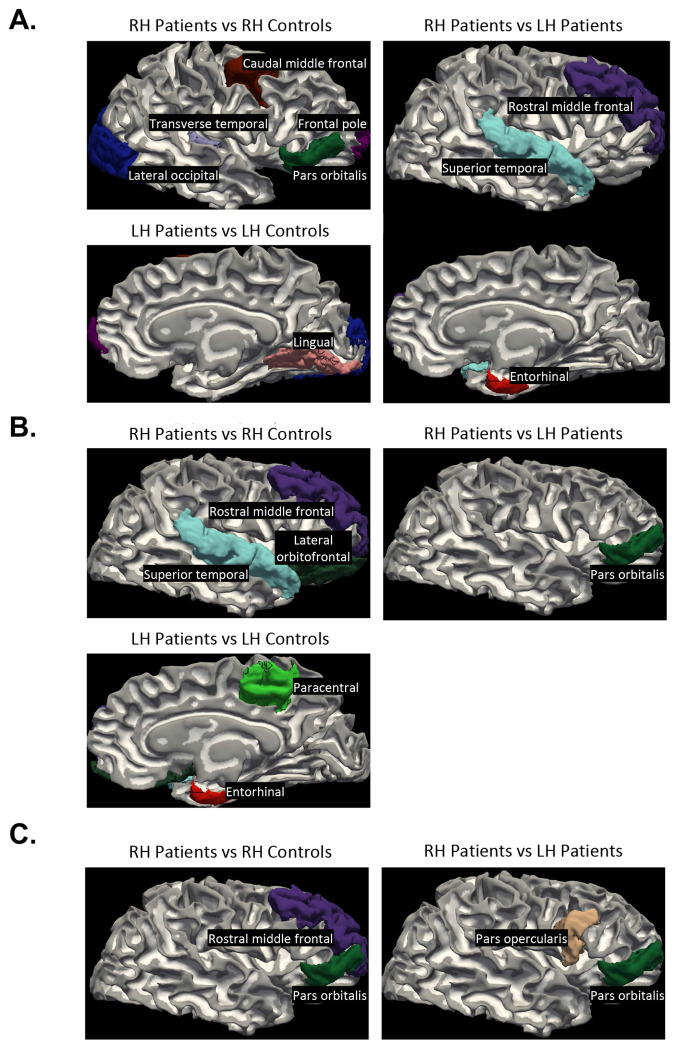
Regions that significantly distinguished between patients versus controls in left columns, right hemisphere patients versus left hemisphere patients in right columns per binary logistic regression modelling for (A) cortical thickness, (B) cortical surface area, and (C) cortical volume. LH = left hemisphere; RH = right hemisphere.

For an illustration of group differences by individual regions per the logistic regressions, see Figure 3.

### Subcortical Morphometrics

**Figure 4:**
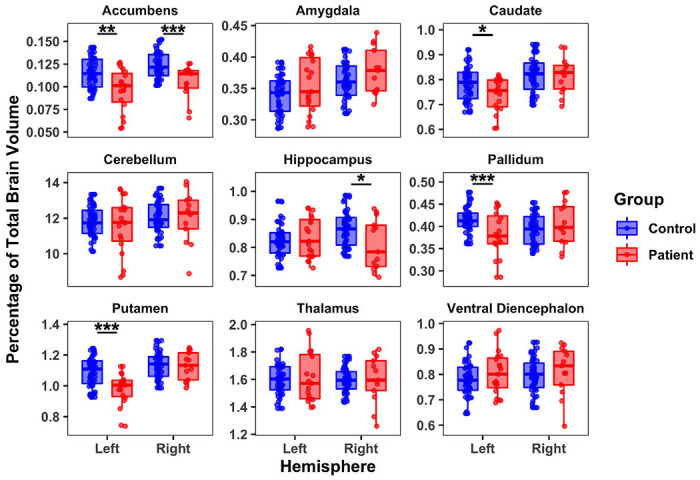
Subcortical regional volumes (normalized) for patients with a preserved left hemisphere or right hemisphere relative to their matched controls *: *p* < .05; **: *p* < .01; ***: *p* < .001.

LH patients had lower accumbens, caudate, pallidum, and putamen volumes relative to controls’ LH. RH patients had lower accumbens and hippocampus volumes relative to controls’ RH. LH patients had lower caudate and putamen volumes than RH patients. See Figure 4 for data and eTables 10-11 for statistics.)

Forward logistic regression analysis yielded a model with putamen and accumbens volumes that could differentiate LH patients vs. LH controls (*r*^2^=0.44). A model with accumbens volume differentiated RH patients vs. RH controls (*r*^2^=0.28). A model with putamen volume differentiated LH from RH patients (*r*^2^=0.52). A regression model that included the putamen volume alone differentiated strongly between the preserved RH vs. LH patients, with *r*^2^=0.52. No predictor/s could differentiate patients’ ILAE outcome scales.

### Analysis Excluding Ablation Cases

One possible explanation for differences between LH vs. RH patients may be because of differences in the extent of the resection, with more LH than RH patients having an ablation: LH – 7 ablation, 12 resection; RH – 2 ablation, 11 resection. Considering this, we recomputed all forward logistic regressions with just the resection cases (12 LH, 11 RH). Notwithstanding loss of statistical power with this reduction, the findings using the three gross measures (LV, GM, and WM) largely mirrored that of the analysis including all patients: whereas no model could predict group membership between LH patients and LH controls or between LH patients and RH patients, a model with LV and GM discriminated between RH patients and RH controls (*r*^2^ = 0.48).

Additionally, cortical analysis with just the resection patients revealed largely similar results to that conducted with all patients. LH patients were differentiated from LH controls with CxT of fusiform and inferior parietal regions as predictors (*r*^2^=0.27). A stronger association between group membership (patient vs. control) for the RH was observed with CxT of frontal pole and posterior cingulate regions as predictors (*r*^2^=.40), and a model with caudal middle frontal and lateral thickness predicted differentiated the two patient groups (*r*^2^=.77). For CSA, no model distinguished between LH patients vs. LH controls. However, a model with lateral orbito-frontal, parahippocampal, rostral middle frontal, and superior temporal CSA differentiated RH patients vs. RH controls (*r*^2^=0.76), and a model with pars orbitalis CSA differentiated the two patients groups (*r*^2^=0.90). The findings of differences in CV were also well-substantiated in the no-ablation analysis. For CV, no model distinguished between LH patients vs. LH controls. Nevertheless, a model with pars orbitalis and rostral middle frontal differentiated RH patients vs RH controls (*r*^2^=0.54), and a model with pars opercularis CV differentiated the two patient groups (*r*^2^=0.70),

Last, subcortical analysis with resection patients replicated the analysis with all patients. A model with accumbens and putamen volumes differentiated LH patients from LH controls (*r*^2^=0.56); accumbens volume differentiated RH patients from RH controls (*r*^2^=0.33); and putamen volume differentiated the two patient groups (*r*^2^=0.66).

## Discussion

Prior morphometry studies, primarily carried out in adults with temporal lobe epilepsy, have typically studied one variable (e.g., CxT or CV) and have also yielded mixed findings^2-5, 37^. Here, we comprehensively characterized the structural integrity of the preserved hemisphere in DRE patients who underwent surgery in *childhood*, a time of greater plasticity than in adulthood ^38^.

We recruited 32 patients, with surgical interventions ranging from large resections (e.g., hemispherectomy) to focal procedures (e.g., ablation), and compared their preserved hemisphere to that of matched controls, and also compared between patient groups. Using GLM and binary logistic regression, we examined gross indices of LV, GM, and WM volumes; multiple indices of 34 cortical parcels; and nine subcortical regions’ volumes. Several findings emerged, summarized below and in Table 1.

First, both patient groups had larger LVs and lower total WM (and GM in RH patients). GM volume alone predicted whether the LH vs. RH was preserved, with less LH than RH atrophy. Second, we quantified CxT, CSA, and CV of 34 cortical regions. GLM showed that LH patients’ measures were not different from controls’, and forward logistic regressions showed the groups were only differentiated by superior temporal region CSA. RH patients differed from controls on all measures in both GLM and forward logistic regression analyses. Between patient groups, differences in pars orbitalis and pars opercularis CSA and CV manifested as key sites in both analyses. Of subcortical regions, accumbens, caudate, hippocampus, pallidum, and putamen volumes were lower in patients vs. controls, and putamen volume alone (smaller in LH than RH) differentiated the two patient groups.

In summary, we identified differences between patients’ preserved RH and each controls’ RH and patients’ LH (and nearly no differences between LH patients vs. LH controls). The preserved RH has lower CxT and CV but, surprisingly, greater CSA in several regions.

Because the two patient groups were matched on age, gender, initial surgery age, seizure-onset age, and ILAE scores, and age and gender were included as covariates in all GLM analyses, none of these factors accounted for hemispheric differences. Both patient groups were roughly equally represented on each scanner and data were harmonized across scanners. Gross replication of results with sub-analyses of only resection cases also rules out this potential confound. Hemispheric differences are also not obviously explained by differences in lobar resections: both LH and RH groups had four temporal resections and two frontal resections. The LH and RH groups had one and two occipital resections, and two and one parietal resections, respectively (number of cases exceed 32 as some patients have multi-lobar resections).

We observed LH vs RH patients’ regional differences primarily in the pars orbitalis and pars triangularis, but also in caudal middle and superior frontal and middle, superior, and transverse temporal regions. These findings, derived from an unbiased, whole-brain, data-driven analysis appear to converge largely on regions implicated in language functions. Resection of the LH, typically the native dominant language hemisphere, is likely a source of alterations of LH homologues of language areas in the preserved RH. In contrast, RH resection results in remarkably few morphometric LH differences relative to controls’ LH, consistent with RH resection children showing better cognitive and language outcomes.^39^

The hypothetical account, then, is that after LH resection, regions of the preserved RH that are homotopic with LH language regions come to assume language functions. The process of accommodating language in the RH (which has some nascent language function^40, 41^) potentially triggers morphometric RH changes. Much evidence attests to the fact that, in early childhood, both hemispheres are predisposed to language function. For example, in contrast to the marked aphasias evident in adults with brain injury, many children with unilateral brain injury have unaffected language function, independent of affected hemisphere.^42^ Likewise, adolescents and young adults who suffered a LH perinatal ischemic stroke displayed sentence-processing abilities comparable to controls, presumably due to RH language engagement.^41, 43^ The idea of RH plasticity for language has also been observed with neuroimaging: individuals with peri- or prenatal periventricular damage to the LH evince RH blood-oxygen-level-dependent activation during a silent word generation task, and the activation extent equaled that of the LH in controls.^44^ These findings attest to the potential for functional reorganization of language to RH homotopic frontotemporal regions.

What remains to be explained is why we see reduction in RH homotopic language regions rather than maintenance or even expansion, which is often associated with the assumption of a new function. The cortical thinning we observe is widespread but, notably, primarily homotopic with or proximal to standard LH language regions (e.g., lateral orbitofrontal, middle temporal, rostral middle frontal, superior frontal, and transverse temporal areas). Presumably, as is also true over the course of normal development, cortical regions are pruned through the removal of inefficient synapses as functions are acquired and the cortical tissue loss reflects improved neural processing by optimizing brain circuits for particular operations.^45^ Cortical thinning is coupled with morphological changes,^45^ and, in childhood, GM thinning,^46^ specifically, is associated with changes in behavior, which may be associated with increases in WM.^47^ In a longitudinal study, changes of CxT asymmetry in the inferior frontal gyrus, constituting a portion of Broca’s area in the LH, is suggestive of a neural correlate of language improvement in children’s brains at roughly ages 5-7 yr.^48^ Furthermore, changes to regions proximal to RH homotopic language areas observed here may reflect the imprecision of the changes (for example, in pruning of neurons and associated dendrites).

Given the cross-sectional, rather than longitudinal, design of the current study, we are unable to evaluate changes over time. Whether the profiles of the childhood DRE brains improved specifically over presurgical profiles or not and whether the relationship between these changes over time, hemisphere of resection, and cognition (specifically language) remain unaddressed and serve as rich fodder for future studies.

## Supplementary Material

Supplement 1

## Figures and Tables

**Table 1:** Summary of all results for univariate and multivariate analyses

Measures	LH patients/LH controls	RH patients/RH controls	RH patients/LH patients
**Gross volumes**			
**General linear model**			
Lateral Ventricle	patients > controls	patients > controls	n.s.
Grey Matter	n.s.	patients < controls	RH < LH
White Matter	patients < controls	patients < controls	n.s.
**Logistic regression**	LV and WM	GM and LV	GM
**Cortical**			
**Thickness**			
**General linear model**	n.s.	patients < controls: caudal middle frontal, cuneus, frontal pole, fusiform, insula, isthmus, lateral orbitofrontal, lingual, middle temporal, paracentral, posterior cingulate, precuneus, rostral middle frontal, superior frontal, transverse temporal	RH < LH: caudal middle frontal, rostral middle frontal, superior frontal
**Logistic regression**	n.s.	caudal middle frontal, frontal pole, lateral occipital, lingual, pars orbitalis, transverse temporal	entorhinal, rostral middle frontal, superior temporal
**Surface area**			
**General linear model**	n.s.	patients > controls: lateral orbitofrontal, parahippocampal, paracentral	LH > RH: pars opercularis, rostral anterior cingulate, transverse temporal; RH > LH: frontal pole, inferior parietal, parahippocampal, pars orbitalis
**Logistic regression**	superior temporal	entorhinal, lateral orbitofrontal, paracentral, rostral middle frontal, superior temporal	pars orbitalis
**Volume**			
**General linear model**	n.s.	patients < controls: rostral middle frontal area	LH > RH: pars opercularis, rostral anterior cingulate, superior frontal, transverse temporal; RH > LH: inferior parietal, pars orbitalis
**Logistic regression**	n.s.	rostral middle frontal, pars orbitalis	pars opercularis, pars orbitalis areas
**Subcortical volumes**			
**General linear model**	patients < controls; accumbens, caudate, pallidum, putamen	patients < controls: accumbens, hippocampus	RH > LH: caudate, putamen
**Logistic regression**	accumbens, putamen	accumbens	putamen

n.s.: not significant

LH: left hemisphere

RH: right hemisphere

## Data Availability

A DOI is reserved on Carnegie Mellon University’s KiltHub (Figshare) repository: 10.1184/R1/24153423. Data/code will be published upon article publication.

## Key Points

### Question:

Following large pediatric cortical resection for the treatment of drug-resistant epilepsy, how does the structural integrity of the preserved hemisphere differ from that of healthy children?

### Findings:

In this case-control study of 32 patients with childhood epilepsy surgery, left-sided—but not right-sided—resection cases showed significantly reduced cortical volume and thickness and increased surface area relative to 51 non-neurological matched controls.

### Meaning:

There is reorganization of the right hemisphere that is specific to left hemispheric resection but not vice versa; this is potentially a consequence of unique left-lateralized functions (e.g., language) requiring postoperative remapping to the right hemisphere.
