# Supplementary material for: Morphometrics of the preserved post-surgical hemisphere in pediatric drug-resistant epilepsy
Source: bioRxiv. 2024 Jan 3:2023.09.24.559189. Originally published 2023 Sep 25. Preprint. [Version 3] doi: 10.1101/2023.09.24.559189 (PMC10557613; doi:10.1101/2023.09.24.559189)
Supplement: Supplement 1 [file media-1.pdf]

eTable 1 Patient information

Age (yr)	Gender	Age at surgery (yr)	Age of seizure onset (yr)	ILAE Scale (Binned)	Surgery
**Left Surgery Patients/Preserved Right**					
11.5	Male	1	0	Low	Hemispherectomy
12.5	Male	0	-	Low	Evacuation of temporal hematoma
13.9	Female	13	6	Low	Occipital and parietal lobectomy
14.7	Male	13	12	Low	Temporal lobectomy with preservation of medial structures & gross total resection of enhancing medial temporal lobe tumor
15.1	Female	14	8	Low	Frontal lobe resection
15.6	Female	13	6	Low	Functional hemispherectomy
15.7	Male	15	7	Low	Frontal lesionectomy with corticectomy
16.1	Male	10	0	High	Temporal lobectomy with amygdalohippocampectomy
16.7	Female	16	13	High	Robot-assisted stereotactic laser ablation of mesial temporal lobe including amygdala and hippocampus (x2)
16.8	Male	4	0	Low	Functional hemispherectomy
19.8	Female	4	1	Low	Hemidecortication
20.5	Male	18	13	Low	Resection of T1 gyrus & Heschl gyrus seizure onset zone
22.2	Female	17	11	Low	Amygdalohippocampectomy, anterior temporal lobectomy, & resection of mesial temporal lobe tumor
**Right Surgery Patients/Preserved Left**					
7.8	Female	3	2	Low	Hemispherotomy
7.8	Male	6	4	Low	Occipital lobectomy, posterior temporal lobectomy, & resection of medial inferior temporal lobe tumor
10.9	Female	10	4	High	Robot-assisted stereotactic laser thermal ablation of frontal seizure onset zone
11.3	Male	10	6	High	Robot-assisted stereotactic laser thermal ablation of amygdala & hippocampus
11.6	Female	4	4	High	Temporal lobe lobectomy with amygdalohippocampectomy
13.1	Female	12	10	High	Robot-assisted stereotactic laser thermal ablation of operculum
14.4	Female	13	3	High	Robot-assisted stereotactic laser thermal ablation of amygdalar seizure onset zone
14.8	Female	8	0	Low	Functional hemispherectomy
15.0	Female	10	9	Low	Resection of frontal operculum, frontal pole, & inferior frontal lobe
15.4	Male	15	4	High	Robot-assisted stereotactic laser thermal ablation of mesial temporal lesion
16.1	Female	1	0	Low	Hemispherotomy
16.6	Male	16	11	Low	Robot-assisted stereotactic laser ablation of occipito-temporal seizure onset zone (x2)
17.0	Female	15	13	Low	Anterior temporal lobectomy & hippocampectomy
17.4	Female	15	10	Low	Resection of parietal lobe, premotor & supplementary motor area
17.8	Male	17	16	Low	Gross total resection of frontal brain tumor
18.3	Male	12	7	Low	Parietal lobectomy & posterior temporal lobectomy
18.7	Female	9	0	Low	Hemispherectomy
19.4	Male	14	6	High	Partial resection of parietooccipital tumor (x2)
20.1	Male	18	10	Low	Robot-assisted stereotactic laser thermal ablation of insulo opercular cortex

Note: Classes 1-3 are binned into the "Low" ILAE scale (better outcome) and Classes 4-6 are binned into the "High" scale (poorer outcome).

eTable 2 R packages

Package	Version	Use
car[1]	3.0-11	Summarizing statistics
DescTools[2]	0.99.44	Data quality control
lme4[3]	1.1-27.1	Fitting linear mixed models
neuroCombat[4]	1.0.9	Harmonization
psych[5]	2.1.9	Computing descriptive statistics
stringr[6]	1.4.0	Data tidying
tidyverse[7]	1.3.1	Data tidying

eTable 3 Descriptive statistics: Median (median absolute deviation) of gross morphometrics

	Control LH	Control RH	Patient LH	Patient RH
LV	5.36x10^3 (1.67x10^3)	4.97x10^3 (1.60x10^3)	6.33x10^3 (3.52x10^3)	5.65x10^3 (3.81x10^3)
GM	2.83x10^5 (2.59x10^4)	2.84x10^5 (2.32x10^4)	2.68x10^5 (3.03x10^4)	2.44x10^5 (2.77x10^4)
WM	2.17x10^5 (2.87x10^4)	2.16x10^5 (2.92x10^4)	2.08x10^5 (2.53x10^4)	1.99x10^5 (2.56x10^4)

LH = left hemisphere
RH = right hemisphere
LV = lateral ventricle
GM = gray matter
WM = white matter

eTable 4 Descriptive statistics: Median cortical thickness (Median absolute deviation) per cortical region

Region	Control LH	Control RH	Patient LH	Patient RH
Banks of Superior Temporal Sulcus	2.65 (1.92x10^-1)	2.78 (2.17x10^-1)	2.59 (9.72x10^-2)	2.68 (2.73x10^-1)
Caudal Anterior Cingulate	2.56 (1.98x10^-1)	2.48 (1.54x10^-1)	2.51 (3.12x10^-1)	2.42 (2.45x10^-1)
Caudal Middle Frontal	2.68 (1.57x10^-1)	2.63 (1.38x10^-1)	2.69 (1.35x10^-1)	2.46 (1.92x10^-1)
Cuneus	2.05 (1.46x10^-1)	2.08 (1.72x10^-1)	1.98 (1.38x10^-1)	1.98 (1.33x10^-1)
Entorhinal	3.17 (2.80x10^-1)	3.13 (4.66x10^-1)	3.01 (2.36x10^-1)	3.25 (4.93x10^-1)
Frontal Pole	2.91 (3.22x10^-1)	2.95 (1.96x10^-1)	2.83 (1.76x10^-1)	2.67 (3.05x10^-1)
Fusiform	2.87 (1.02x10^-1)	2.89 (1.07x10^-1)	2.83 (1.46x10^-1)	2.79 (9.55x10^-2)
Inferior Parietal	2.67 (1.43x10^-1)	2.73 (1.63x10^-1)	2.62 (1.25x10^-1)	2.6 (1.00x10^-1)
Inferior Temporal	2.94 (1.34x10^-1)	2.99 (1.61x10^-1)	2.84 (1.42x10^-1)	2.92 (1.38x10^-1)
Insula	3.1 (1.52x10^-1)	3.12 (1.72x10^-1)	3.09 (1.79x10^-1)	3.02 (1.71x10^-1)
Isthmus	2.42 (1.58x10^-1)	2.48 (2.05x10^-1)	2.41 (1.99x10^-1)	2.31 (2.12x10^-1)
Lateral Occipital	2.3 (1.39x10^-1)	2.4 (1.17x10^-1)	2.3 (1.10x10^-1)	2.28 (1.19x10^-1)
Lateral Orbitofrontal	2.75 (1.59x10^-1)	2.74 (1.09x10^-1)	2.74 (1.78x10^-1)	2.67 (1.58x10^-1)
Lingual	2.18 (1.37x10^-1)	2.17 (8.76x10^-2)	2.15 (1.69x10^-1)	2.03 (1.75x10^-1)
Medial Orbitofrontal	2.53 (1.21x10^-1)	2.57 (1.85x10^-1)	2.57 (1.80x10^-1)	2.49 (7.38x10^-2)
Middle Temporal	3.01 (1.43x10^-1)	3.05 (1.39x10^-1)	3.01 (1.24x10^-1)	2.94 (1.99x10^-1)
Parahippocampal	2.64 (1.27x10^-1)	2.59 (2.02x10^-1)	2.64 (1.65x10^-1)	2.57 (1.38x10^-1)
Paracentral	2.85 (2.67x10^-1)	2.8 (1.99x10^-1)	2.78 (3.91x10^-1)	2.59 (2.41x10^-1)
Pars Opercularis	2.8 (1.46x10^-1)	2.77 (1.49x10^-1)	2.68 (1.13x10^-1)	2.59 (2.21x10^-1)
Pars Orbitalis	2.84 (1.95x10^-1)	2.83 (2.64x10^-1)	2.81 (1.37x10^-1)	2.68 (3.19x10^-1)
Pars Triangularis	2.63 (2.12x10^-1)	2.62 (1.58x10^-1)	2.61 (1.83x10^-1)	2.36 (2.35x10^-1)
Pericalcarine	1.73 (1.55x10^-1)	1.75 (1.61x10^-1)	1.73 (9.06x10^-2)	1.64 (1.63x10^-1)
Postcentral	2.27 (1.53x10^-1)	2.22 (1.73x10^-1)	2.26 (1.57x10^-1)	2.23 (3.08x10^-1)
Posterior Cingulate	2.54 (1.32x10^-1)	2.5 (1.79x10^-1)	2.52 (1.62x10^-1)	2.42 (2.08x10^-1)
Precentral	2.72 (1.28x10^-1)	2.65 (1.77x10^-1)	2.71 (1.46x10^-1)	2.56 (1.68x10^-1)
Precuneus	2.62 (1.02x10^-1)	2.63 (1.51x10^-1)	2.58 (1.05x10^-1)	2.51 (1.53x10^-1)
Rostral Anterior Cingulate	2.89 (1.86x10^-1)	2.79 (2.27x10^-1)	2.87 (2.64x10^-1)	2.82 (1.28x10^-1)
Rostral Middle Frontal	2.53 (1.38x10^-1)	2.42 (1.35x10^-1)	2.51 (1.35x10^-1)	2.3 (1.95x10^-1)

Superior Frontal	2.85 (1.70x10^-1)	2.78 (1.57x10^-1)	2.83 (1.41x10^-1)	2.61 (2.19x10^-1)
Superior Parietal	2.41 (1.50x10^-1)	2.4 (1.37x10^-1)	2.38 (1.46x10^-1)	2.33 (1.57x10^-1)
Superior Temporal	3 (1.06x10^-1)	3 (1.60x10^-1)	2.96 (1.24x10^-1)	2.92 (1.65x10^-1)
Supramarginal	2.75 (1.14x10^-1)	2.77 (1.94x10^-1)	2.73 (1.38x10^-1)	2.62 (1.67x10^-1)
Temporal Pole	3.42 (2.00x10^-1)	3.51 (4.05x10^-1)	3.37 (2.97x10^-1)	3.5 (5.69x10^-1)
Transverse Temporal	2.66 (2.07x10^-1)	2.7 (1.95x10^-1)	2.53 (1.49x10^-1)	2.5 (3.54x10^-1)

LH = left hemisphere
RH = right hemisphere

eTable 5 General linear model results: Cortical thickness

**Region**	**Comparison**	***p*-value**	***Bayes factor***
Banks of superior temporal sulcus	LC vs. LP	.99	8.33
	RC vs. RP	.15	2.16
	LP vs. RP	.40	2.81
Caudal anterior cingulate	LC vs. LP	.99	8.32
	RC vs. RP	.96	7.99
	LP vs. RP	.29	1.65
Caudal middle frontal	LC vs. LP	.99	8.30
	RC vs. RP	**.02**	4.61x10^-2
	LP vs. RP	**.01**	1.01x10^-2
Cuneus	LC vs. LP	.99	8.34
	RC vs. RP	**.02**	1.05x10^-1
	LP vs. RP	.29	2.24
Entorhinal	LC vs. LP	.99	4.75
	RC vs. RP	.82	7.63
	LP vs. RP	.40	2.84
Frontal pole	LC vs. LP	.99	6.88
	RC vs. RP	**.02**	2.35x10^-2
	LP vs. RP	.18	5.53x10^-1
Fusiform	LC vs. LP	.99	9.48x10^-1
	RC vs. RP	**.02**	4.92x10^-2
	LP vs. RP	.55	4.45
Inferior parietal	LC vs. LP	.99	8.15
	RC vs. RP	.15	1.86
	LP vs. RP	.76	5.36
Inferior temporal	LC vs. LP	.99	2.83
	RC vs. RP	.07	6.62x10^-1
	LP vs. RP	.91	5.61
Insula	LC vs. LP	.99	6.78
	RC vs. RP	**.03**	3.43x10^-1
	LP vs. RP	.40	3.24
Isthmus	LC vs. LP	.99	7.24
	RC vs. RP	**.02**	8.85x10^-2
	LP vs. RP	.22	8.98x10^-1
Lateral occipital	LC vs. LP	.99	7.11
	RC vs. RP	.49	5.31
	LP vs. RP	.52	4.00
Lateral orbitofrontal	LC vs. LP	.99	7.73
	RC vs. RP	**.02**	1.36x10^-1
	LP vs. RP	.22	1.02
Lingual	LC vs. LP	.99	3.23
	RC vs. RP	**.02**	5.65x10^-2
	LP vs. RP	.34	2.20

Medial orbitofrontal	LC vs. LP	.99	8.04
	RC vs. RP	.13	1.65
	LP vs. RP	.18	5.00x10^-1
Middle temporal	LC vs. LP	.99	8.07
	RC vs. RP	**.03**	3.14x10^-1
	LP vs. RP	.40	3.27
Parahippocampal	LC vs. LP	.99	4.50
	RC vs. RP	.06	5.36x10^-1
	LP vs. RP	.29	1.69
Paracentral	LC vs. LP	.99	4.53
	RC vs. RP	**.03**	3.43x10^-1
	LP vs. RP	.26	1.38
Pars opercularis	LC vs. LP	.99	1.39
	RC vs. RP	.06	5.12x10^-1
	LP vs. RP	.19	6.85x10^-1
Pars orbitalis	LC vs. LP	.99	5.07
	RC vs. RP	.95	7.95
	LP vs. RP	.55	4.27
Pars triangularis	LC vs. LP	.99	8.07
	RC vs. RP	.07	8.04x10^-1
	LP vs. RP	.08	1.10x10^-1
Pericalcarine	LC vs. LP	.99	8.08
	RC vs. RP	.10	1.35
	LP vs. RP	.18	2.66x10^-1
Postcentral	LC vs. LP	.99	7.69
	RC vs. RP	.90	7.84
	LP vs. RP	.40	3.01
Posterior cingulate	LC vs. LP	>.99	8.37
	RC vs. RP	**.02**	2.00x10^-1
	LP vs. RP	.18	5.27x10^-1
Precentral	LC vs. LP	.99	5.16
	RC vs. RP	.28	3.19
	LP vs. RP	.22	1.04
Precuneus	LC vs. LP	.99	5.60
	RC vs. RP	**.02**	1.61x10^-1
	LP vs. RP	.18	5.69x10^-1
Rostral anterior cingulate	LC vs. LP	.99	5.12
	RC vs. RP	.69	6.96
	LP vs. RP	.29	1.81
Rostral middle frontal	LC vs. LP	.99	8.20
	RC vs. RP	**.02**	3.75x10^-2
	LP vs. RP	**<.001**	1.06x10^-3
Superior frontal	LC vs. LP	.99	8.13
	RC vs. RP	**.03**	1.84x10^-1
	LP vs. RP	**<.001**	2.65x10^-3
Superior parietal	LC vs. LP	.99	8.15
	RC vs. RP	.14	1.94
	LP vs. RP	.29	1.88
Superior temporal	LC vs. LP	.99	8.05
	RC vs. RP	.28	3.24
	LP vs. RP	.50	3.94
Supramarginal	LC vs. LP	.99	5.66
	RC vs. RP	.07	6.96x10^-1

	LP vs. RP	.22	8.24x10^-1
Temporal pole	LC vs. LP	>.99	8.37
	RC vs. RP	.69	6.90
	LP vs. RP	.40	3.09
Transverse temporal	LC vs. LP	.99	7.45
	RC vs. RP	**.02**	1.50x10^-2
	LP vs. RP	.22	1.08

LC = left hemisphere of controls
LP = left hemisphere of patients
RC = right hemisphere of controls
RP = right hemisphere of patients
p-values less than .05 are bolded.

eTable 6 Descriptive statistics: Median normalized cortical surface area (median absolute deviations per cortical area

Region	Control LH	Control RH	Patient LH	Patient RH
Banks of Superior Temporal Sulcus	4.13x10^-1 (5.34x10^-2)	3.65x10^-1 (3.72x10^-2)	4.08x10^-1 (5.47x10^-2)	3.48x10^-1 (5.92x10^-2)
Caudal Anterior Cingulate	2.35x10^-1 (4.14x10^-2)	2.99x10^-1 (4.80x10^-2)	2.39x10^-1 (4.03x10^-2)	2.54x10^-1 (7.73x10^-2)
Caudal Middle Frontal	8.51x10^-1 (7.57x10^-2)	7.82x10^-1 (1.30x10^-1)	9.04x10^-1 (1.24x10^-1)	7.61x10^-1 (1.27x10^-1)
Cuneus	6.34x10^-1 (8.12x10^-2)	6.56x10^-1 (6.47x10^-2)	6.13x10^-1 (6.46x10^-2)	6.75x10^-1 (5.51x10^-2)
Entorhinal	1.70x10^-1 (2.21x10^-2)	1.59x10^-1 (3.39x10^-2)	1.52x10^-1 (3.27x10^-2)	1.62x10^-1 (1.98x10^-2)
Frontal Pole	1.14x10^-1 (1.05x10^-2)	1.42x10^-1 (1.51x10^-2)	1.13x10^-1 (9.08x10^-3)	1.43x10^-1 (2.01x10^-2)
Fusiform	1.23 (9.00x10^-2)	1.24 (1.09x10^-1)	1.26 (1.53x10^-1)	1.31 (8.90x10^-2)
Inferior Parietal	1.82 (1.86x10^-1)	2.14 (2.24x10^-1)	1.79 (1.42x10^-1)	2.2 (2.73x10^-1)
Inferior Temporal	1.39 (1.48x10^-1)	1.31 (1.19x10^-1)	1.4 (1.93x10^-1)	1.37 (9.34x10^-2)
Insula	8.57x10^-1 (5.92x10^-2)	8.27x10^-1 (6.29x10^-2)	8.97x10^-1 (9.83x10^-2)	8.73x10^-1 (6.95x10^-2)
Isthmus	4.10x10^-1 (5.17x10^-2)	3.67x10^-1 (3.49x10^-2)	4.12x10^-1 (6.16x10^-2)	3.76x10^-1 (4.60x10^-2)
Lateral Occipital	2.02 (2.10x10^-1)	2.1 (1.76x10^-1)	2.01 (1.80x10^-1)	2.05 (2.31x10^-1)
Lateral Orbitofrontal	1.07 (6.76x10^-2)	1.02 (8.41x10^-2)	1.07 (9.37x10^-2)	1.09 (7.28x10^-2)
Lingual	1.25 (1.36x10^-1)	1.34 (1.52x10^-1)	1.26 (1.18x10^-1)	1.32 (1.57x10^-1)
Medial Orbitofrontal	7.58x10^-1 (5.75x10^-2)	8.13x10^-1 (4.96x10^-2)	7.75x10^-1 (5.79x10^-2)	8.52x10^-1 (4.46x10^-2)
Middle Temporal	1.33 (8.11x10^-2)	1.45 (8.40x10^-2)	1.35 (7.67x10^-2)	1.41 (4.05x10^-2)
Parahippocampal	5.01x10^-1 (4.98x10^-2)	5.65x10^-1 (4.66x10^-2)	5.09x10^-1 (6.97x10^-2)	5.99x10^-1 (1.05x10^-1)
Paracentral	2.49x10^-1 (2.69x10^-2)	2.37x10^-1 (2.53x10^-2)	2.42x10^-1 (3.98x10^-2)	2.59x10^-1 (2.92x10^-2)
Pars Opercularis	6.51x10^-1 (8.54x10^-2)	5.27x10^-1 (5.57x10^-2)	6.52x10^-1 (5.83x10^-2)	5.06x10^-1 (4.28x10^-2)
Pars Orbitalis	2.88x10^-1 (1.80x10^-2)	3.43x10^-1 (3.24x10^-2)	2.82x10^-1 (2.71x10^-2)	3.55x10^-1 (2.40x10^-2)
Pars Triangularis	5.60x10^-1 (6.30x10^-2)	6.19x10^-1 (9.88x10^-2)	5.40x10^-1 (7.78x10^-2)	6.07x10^-1 (8.17x10^-2)
Pericalcarine	5.73x10^-1 (8.84x10^-2)	6.31x10^-1 (9.16x10^-2)	5.77x10^-1 (9.78x10^-2)	6.56x10^-1 (8.94x10^-2)
Postcentral	1.57 (1.38x10^-1)	1.5 (1.62x10^-1)	1.56 (1.12x10^-1)	1.55 (1.88x10^-1)
Posterior Cingulate	4.64x10^-1 (4.12x10^-2)	4.76x10^-1 (5.24x10^-2)	4.95x10^-1 (5.13x10^-2)	4.78x10^-1 (6.62x10^-2)
Precentral	1.81 (1.18x10^-1)	1.81 (2.23x10^-1)	1.82 (1.57x10^-1)	1.8 (1.28x10^-1)
Precuneus	1.5 (1.06x10^-1)	1.57 (1.40x10^-1)	1.56 (1.32x10^-1)	1.52 (1.08x10^-1)
Rostral Anterior Cingulate	3.23x10^-1 (6.06x10^-2)	2.47x10^-1 (3.67x10^-2)	3.24x10^-1 (4.39x10^-2)	2.42x10^-1 (8.91x10^-2)
Rostral Middle Frontal	2.33 (1.90x10^-1)	2.41 (1.52x10^-1)	2.27 (1.65x10^-1)	2.23 (2.86x10^-1)
Superior Frontal	2.83 (1.49x10^-1)	2.72 (2.39x10^-1)	2.91 (2.96x10^-1)	2.65 (2.26x10^-1)

Superior Parietal	2.13 (2.33x10^-1)	2.05 (2.65x10^-1)	2.23 (2.64x10^-1)	2.11 (1.45x10^-1)
Superior Temporal	1.58 (9.30x10^-2)	1.45 (1.02x10^-1)	1.53 (1.12x10^-1)	1.42 (1.35x10^-1)
Supramarginal	1.54 (1.96x10^-1)	1.42 (1.72x10^-1)	1.53 (2.29x10^-1)	1.57 (1.24x10^-1)
Temporal Pole	2.01x10^-1 (2.81x10^-2)	1.93x10^-1 (1.84x10^-2)	1.97x10^-1 (1.66x10^-2)	2.06x10^-1 (2.80x10^-2)
Transverse Temporal	1.76x10^-1 (1.55x10^-2)	1.30x10^-1 (1.17x10^-2)	1.71x10^-1 (2.63x10^-2)	1.27x10^-1 (2.51x10^-2)

LH = left hemisphere
RH = right hemisphere

eTable 7 General linear model results: Cortical surface area

Region	Comparison	*p*-value	***Bayes factor***
Banks of superior temporal sulcus	LC vs. LP	.54	0.98
	RC vs. RP	.87	5.79
	LP vs. RP	.10	3.59x10^-1
Caudal anterior cingulate	LC vs. LP	.95	7.93
	RC vs. RP	.29	1.26
	LP vs. RP	.77	4.68
Caudal middle frontal	LC vs. LP	.95	7.31
	RC vs. RP	.91	7.86
	LP vs. RP	.31	1.45
Cuneus	LC vs. LP	.95	8.28
	RC vs. RP	.87	6.16
	LP vs. RP	.39	2.28
Entorhinal	LC vs. LP	.54	1.82
	RC vs. RP	.92	7.96
	LP vs. RP	.97	5.65
Frontal pole	LC vs. LP	.54	1.61
	RC vs. RP	.87	7.44
	LP vs. RP	**<.001**	4.82x10^-6
Fusiform	LC vs. LP	.95	7.72
	RC vs. RP	.87	7.05
	LP vs. RP	.87	5.40
Inferior parietal	LC vs. LP	.81	5.73
	RC vs. RP	.91	7.70
	LP vs. RP	**<.001**	4.62x10^-3
Inferior temporal	LC vs. LP	.86	6.62
	RC vs. RP	.87	7.39
	LP vs. RP	.39	2.30
Insula	LC vs. LP	.86	6.49
	RC vs. RP	.87	5.74
	LP vs. RP	.77	4.53
Isthmus	LC vs. LP	.81	4.85
	RC vs. RP	.87	7.31
	LP vs. RP	.09	2.61x10^-1
Lateral occipital	LC vs. LP	.97	8.36
	RC vs. RP	.87	6.35
	LP vs. RP	.87	5.43
Lateral orbitofrontal	LC vs. LP	.95	8.23
	RC vs. RP	**.02**	4.88x10^-2
	LP vs. RP	.49	2.92
Lingual	LC vs. LP	.95	8.21
	RC vs. RP	.91	7.84
	LP vs. RP	.61	3.54

Medial orbitofrontal	LC vs. LP	.58	2.35
	RC vs. RP	.87	6.54
	LP vs. RP	.19	6.95x10^-1
Middle temporal	LC vs. LP	.81	5.63
	RC vs. RP	.73	4.06
	LP vs. RP	.17	6.31x10^-1
Parahippocampal	LC vs. LP	.95	7.54
	RC vs. RP	**.02**	4.39x10^-2
	LP vs. RP	**<.001**	5.38x10^-3
Paracentral	LC vs. LP	.95	8.30
	RC vs. RP	**<.001**	5.88x10^-2
	LP vs. RP	.39	2.30
Pars opercularis	LC vs. LP	.81	6.17
	RC vs. RP	.59	2.87
	LP vs. RP	**<.001**	2.36x10^-5
Pars orbitalis	LC vs. LP	.58	2.60
	RC vs. RP	.29	1.07
	LP vs. RP	**<.001**	5.96x10^-8
Pars triangularis	LC vs. LP	.95	8.18
	RC vs. RP	.70	3.79
	LP vs. RP	.39	2.22
Pericalcarine	LC vs. LP	.95	8.12
	RC vs. RP	.92	7.92
	LP vs. RP	.39	2.01
Postcentral	LC vs. LP	.81	5.36
	RC vs. RP	.87	5.62
	LP vs. RP	.87	5.34
Posterior cingulate	LC vs. LP	.81	5.35
	RC vs. RP	.87	7.43
	LP vs. RP	.87	5.47
Precentral	LC vs. LP	.81	3.76
	RC vs. RP	.87	5.74
	LP vs. RP	.95	5.61
Precuneus	LC vs. LP	.92	7.08
	RC vs. RP	.87	5.54
	LP vs. RP	.87	5.22
Rostral anterior cingulate	LC vs. LP	.81	5.86
	RC vs. RP	.87	7.58
	LP vs. RP	**<.001**	5.14x10^-3
Rostral middle frontal	LC vs. LP	.95	8.06
	RC vs. RP	.14	3.36
	LP vs. RP	.87	5.10
Superior frontal	LC vs. LP	.81	3.89
	RC vs. RP	.87	7.51
	LP vs. RP	.12	4.17x10^-1
Superior parietal	LC vs. LP	.81	4.69
	RC vs. RP	.91	7.82
	LP vs. RP	.69	3.83
Superior temporal	LC vs. LP	.20	8.12x10^-2
	RC vs. RP	.14	5.88x10^-1
	LP vs. RP	.07	1.51x10^-1
Supramarginal	LC vs. LP	.54	1.78
	RC vs. RP	.14	4.18x10^-1
	LP vs. RP	.87	5.28

Temporal pole	LC vs. LP	.97	8.34
	RC vs. RP	.59	2.95
	LP vs. RP	.87	5.47
Transverse temporal	LC vs. LP	.20	4.64x10^-1
	RC vs. RP	.87	6.76
	LP vs. RP	**<.001**	1.42x10^-5

LC = left hemisphere of controls
LP = left hemisphere of patients
RC = right hemisphere of controls
RP = right hemisphere of patients
p-values less than .05 are bolded.

eTable 8 Descriptive statistics: Median normalized region volume (median absolute deviations) per cortical area

Region	Control LH	Control RH	Patient LH	Patient RH
Banks of Superior Temporal Sulcus	5.96x10^-3 (1.10x10^-3)	5.25x10^-3 (7.56x10^-4)	5.58x10^-3 (9.94x10^-4)	4.74x10^-3 (6.36x10^-4)
Caudal Anterior Cingulate	3.71x10^-3 (6.67x10^-4)	4.62x10^-3 (1.01x10^-3)	3.82x10^-3 (9.24x10^-4)	3.89x10^-3 (1.58x10^-3)
Caudal Middle Frontal	1.37x10^-2 (1.50x10^-3)	1.25x10^-2 (2.38x10^-3)	1.45x10^-2 (2.77x10^-3)	1.11x10^-2 (1.37x10^-3)
Cuneus	7.40x10^-3 (1.16x10^-3)	8.04x10^-3 (1.23x10^-3)	7.25x10^-3 (2.17x10^-3)	8.40x10^-3 (6.12x10^-4)
Entorhinal	3.86x10^-3 (5.96x10^-4)	3.71x10^-3 (8.79x10^-4)	3.58x10^-3 (4.69x10^-4)	3.73x10^-3 (1.36x10^-3)
Frontal Pole	2.63x10^-3 (4.52x10^-4)	3.27x10^-3 (4.02x10^-4)	2.44x10^-3 (5.64x10^-4)	2.86x10^-3 (5.00x10^-4)
Fusiform	2.16x10^-2 (1.62x10^-3)	2.29x10^-2 (2.45x10^-3)	2.17x10^-2 (2.95x10^-3)	2.22x10^-2 (2.59x10^-3)
Inferior Parietal	2.96x10^-2 (4.09x10^-3)	3.69x10^-2 (5.81x10^-3)	2.88x10^-2 (3.15x10^-3)	3.75x10^-2 (3.96x10^-3)
Inferior Temporal	2.65x10^-2 (2.97x10^-3)	2.59x10^-2 (3.32x10^-3)	2.62x10^-2 (3.59x10^-3)	2.53x10^-2 (3.01x10^-3)
Insula	1.48x10^-2 (1.03x10^-3)	1.46x10^-2 (1.44x10^-3)	1.50x10^-2 (1.47x10^-3)	1.49x10^-2 (1.52x10^-3)
Isthmus	6.24x10^-3 (6.96x10^-4)	5.76x10^-3 (7.47x10^-4)	6.25x10^-3 (1.41x10^-3)	5.23x10^-3 (6.95x10^-4)
Lateral Occipital	2.87x10^-2 (3.19x10^-3)	3.00x10^-2 (2.83x10^-3)	2.87x10^-2 (2.67x10^-3)	3.05x10^-2 (2.36x10^-3)
Lateral Orbitofrontal	1.79x10^-2 (1.37x10^-3)	1.71x10^-2 (1.23x10^-3)	1.77x10^-2 (1.15x10^-3)	1.71x10^-2 (9.44x10^-4)
Lingual	1.56x10^-2 (2.00x10^-3)	1.68x10^-2 (2.51x10^-3)	1.51x10^-2 (2.95x10^-3)	1.65x10^-2 (2.09x10^-3)
Medial Orbitofrontal	1.18x10^-2 (1.15x10^-3)	1.35x10^-2 (1.18x10^-3)	1.26x10^-2 (8.73x10^-4)	1.30x10^-2 (3.87x10^-4)
Middle Temporal	2.80x10^-2 (3.68x10^-3)	3.03x10^-2 (2.20x10^-3)	2.85x10^-2 (2.30x10^-3)	2.79x10^-2 (1.24x10^-3)
Parahippocampal	8.17x10^-3 (1.07x10^-3)	8.87x10^-3 (7.65x10^-4)	8.09x10^-3 (1.08x10^-3)	8.78x10^-3 (7.56x10^-4)
Paracentral	4.42x10^-3 (6.49x10^-4)	4.22x10^-3 (5.31x10^-4)	4.31x10^-3 (7.23x10^-4)	4.25x10^-3 (2.40x10^-4)
Pars Opercularis	1.12x10^-2 (1.70x10^-3)	8.97x10^-3 (1.05x10^-3)	1.13x10^-2 (1.43x10^-3)	8.28x10^-3 (1.41x10^-3)
Pars Orbitalis	6.00x10^-3 (5.57x10^-4)	6.95x10^-3 (7.75x10^-4)	5.70x10^-3 (1.08x10^-3)	7.09x10^-3 (1.20x10^-3)
Pars Triangularis	9.05x10^-3 (1.24x10^-3)	1.03x10^-2 (2.11x10^-3)	8.96x10^-3 (1.28x10^-3)	9.61x10^-3 (2.15x10^-3)
Pericalcarine	4.67x10^-3 (1.03x10^-3)	5.30x10^-3 (1.17x10^-3)	4.88x10^-3 (1.05x10^-3)	4.92x10^-3 (4.81x10^-4)
Postcentral	2.19x10^-2 (2.52x10^-3)	2.09x10^-2 (1.88x10^-3)	2.27x10^-2 (3.05x10^-3)	2.09x10^-2 (3.52x10^-3)
Posterior Cingulate	7.02x10^-3 (9.60x10^-4)	7.39x10^-3 (7.71x10^-4)	7.04x10^-3 (7.81x10^-4)	6.91x10^-3 (1.18x10^-3)
Precentral	2.93x10^-2 (2.32x10^-3)	2.84x10^-2 (2.95x10^-3)	3.09x10^-2 (3.63x10^-3)	2.79x10^-2 (3.95x10^-3)
Precuneus	2.31x10^-2 (2.53x10^-3)	2.40x10^-2 (2.50x10^-3)	2.38x10^-2 (2.27x10^-3)	2.24x10^-2 (2.60x10^-3)
Rostral Anterior Cingulate	5.94x10^-3 (8.95x10^-4)	4.45x10^-3 (9.42x10^-4)	5.93x10^-3 (8.32x10^-4)	4.79x10^-3 (3.52x10^-4)
Rostral Middle Frontal	3.70x10^-2 (3.57x10^-3)	3.82x10^-2 (4.22x10^-3)	3.57x10^-2 (3.66x10^-3)	3.30x10^-2 (6.44x10^-3)

Superior Frontal	5.22x10^-2 (4.79x10^-3)	4.86x10^-2 (5.25x10^-3)	5.26x10^-2 (7.14x10^-3)	4.42x10^-2 (3.19x10^-3)
Superior Parietal	3.08x10^-2 (5.14x10^-3)	3.04x10^-2 (3.92x10^-3)	3.24x10^-2 (4.33x10^-3)	3.02x10^-2 (2.96x10^-3)
Superior Temporal	3.00x10^-2 (2.53x10^-3)	2.77x10^-2 (2.78x10^-3)	2.94x10^-2 (2.56x10^-3)	2.65x10^-2 (3.12x10^-3)
Supramarginal	2.56x10^-2 (3.74x10^-3)	2.36x10^-2 (3.49x10^-3)	2.57x10^-2 (4.49x10^-3)	2.56x10^-2 (4.01x10^-3)
Temporal Pole	5.23x10^-3 (7.17x10^-4)	5.21x10^-3 (8.66x10^-4)	5.41x10^-3 (1.06x10^-3)	5.88x10^-3 (6.90x10^-4)
Transverse Temporal	2.80x10^-3 (4.28x10^-4)	2.17x10^-3 (2.24x10^-4)	2.61x10^-3 (2.42x10^-4)	1.93x10^-3 (3.26x10^-4)

LH = left hemisphere
RH = right hemisphere

eTable 9 General linear model results: Cortical volume

**Region**	**Comparison**	***p*-value**	***Bayes factor***
Banks of superior temporal sulcus	LC vs. LP	.75	2.61
	RC vs. RP	.61	5.78
	LP vs. RP	.27	1.30
Caudal anterior cingulate	LC vs. LP	.99	8.36
	RC vs. RP	.30	1.64
	LP vs. RP	.84	4.97
Caudal middle frontal	LC vs. LP	.75	5.36
	RC vs. RP	.61	5.66
	LP vs. RP	.07	1.66x10^-1
Cuneus	LC vs. LP	.75	5.67
	RC vs. RP	.84	7.57
	LP vs. RP	.84	4.61
Entorhinal	LC vs. LP	.75	1.16
	RC vs. RP	.85	7.67
	LP vs. RP	.45	2.12
Frontal pole	LC vs. LP	.75	2.81
	RC vs. RP	.30	5.04x10^-1
	LP vs. RP	.07	1.92x10^-1
Fusiform	LC vs. LP	.99	8.33
	RC vs. RP	.53	4.70
	LP vs. RP	.97	5.63
Inferior parietal	LC vs. LP	.75	5.72
	RC vs. RP	.79	7.07
	LP vs. RP	**.01**	2.29x10^-3
Inferior temporal	LC vs. LP	.99	7.77
	RC vs. RP	.79	7.24
	LP vs. RP	.80	3.54
Insula	LC vs. LP	.99	7.51
	RC vs. RP	.83	7.45
	LP vs. RP	.97	5.65
Isthmus	LC vs. LP	.75	5.40
	RC vs. RP	.30	7.72x10^-1
	LP vs. RP	.07	1.83x10^-1
Lateral occipital	LC vs. LP	.99	8.36
	RC vs. RP	.73	6.53
	LP vs. RP	.84	4.72
Lateral orbitofrontal	LC vs. LP	.99	7.39
	RC vs. RP	.30	1.27
	LP vs. RP	.83	4.07
Lingual	LC vs. LP	.77	6.25

	RC vs. RP	.45	3.81
	LP vs. RP	.93	5.37
Medial orbitofrontal	LC vs. LP	.75	4.39
	RC vs. RP	.61	5.56
	LP vs. RP	.12	6.06x10^-1
Middle temporal	LC vs. LP	.75	3.14
	RC vs. RP	.30	1.36
	LP vs. RP	.97	5.64
Parahippocampal	LC vs. LP	.99	8.36
	RC vs. RP	.34	2.77
	LP vs. RP	.07	2.39x10^-1
Paracentral	LC vs. LP	.99	8.34
	RC vs. RP	.34	2.85
	LP vs. RP	.93	5.42
Pars opercularis	LC vs. LP	.75	5.70
	RC vs. RP	.30	2.16
	LP vs. RP	**<.001**	3.55x10^-5
Pars orbitalis	LC vs. LP	.75	3.40
	RC vs. RP	.30	2.43
	LP vs. RP	**<.001**	2.34x10^-4
Pars triangularis	LC vs. LP	.99	8.36
	RC vs. RP	.30	2.05
	LP vs. RP	.93	5.51
Pericalcarine	LC vs. LP	.99	8.36
	RC vs. RP	.54	5.04
	LP vs. RP	.97	5.56
Postcentral	LC vs. LP	.99	7.80
	RC vs. RP	.52	4.49
	LP vs. RP	.84	4.99
Posterior cingulate	LC vs. LP	.99	7.80
	RC vs. RP	.30	1.49
	LP vs. RP	.81	3.80
Precentral	LC vs. LP	.75	2.11
	RC vs. RP	.98	7.99
	LP vs. RP	.26	1.04
Precuneus	LC vs. LP	.77	6.09
	RC vs. RP	.30	1.56
	LP vs. RP	.84	4.48
Rostral anterior cingulate	LC vs. LP	.99	8.37
	RC vs. RP	.98	8.00
	LP vs. RP	**<.001**	5.80x10^-3
Rostral middle frontal	LC vs. LP	.99	8.33
	RC vs. RP	**<.001**	1.49x10^-3
	LP vs. RP	.09	3.07x10^-1
Superior frontal	LC vs. LP	.75	3.52
	RC vs. RP	.30	2.08
	LP vs. RP	**.01**	1.39x10^-2
Superior parietal	LC vs. LP	.75	4.66
	RC vs. RP	.88	7.81
	LP vs. RP	.84	4.20
Superior temporal	LC vs. LP	.75	2.56
	RC vs. RP	.30	1.76
	LP vs. RP	.07	2.31x10^-1
Supramarginal	LC vs. LP	.75	5.50

	RC vs. RP	.30	2.29
	LP vs. RP	.84	4.91
Temporal pole	LC vs. LP	.75	5.20
	RC vs. RP	.30	1.61
	LP vs. RP	.87	5.13
Transverse temporal	LC vs. LP	.75	2.39
	RC vs. RP	.30	6.01x10^-1
	LP vs. RP	**<.001**	1.30x10^-4

LC = left hemisphere of controls
LP = left hemisphere of patients
RC = right hemisphere of controls
RP = right hemisphere of patients
p-values less than .05 are bolded.

eTable 10 Descriptive statistics: Median volume (median absolute deviations) per subcortical area

**Region**	**Control LH**	**Control RH**	**Patient LH**	**Patient RH**
Accumbens	1.14x10^-3 (2.33x10^-4)	1.22x10^-3 (1.97x10^-4)	1.01x10^-3 (2.33x10^-4)	1.14x10^-3 (1.65x10^-4)
Amygdala	3.43x10^-3 (3.95x10^-4)	3.60x10^-3 (3.29x10^-4)	3.45x10^-3 (5.90x10^-4)	3.79x10^-3 (4.80x10^-4)
Caudate	7.89x10^-3 (7.50x10^-4)	8.23x10^-3 (6.95x10^-4)	7.56x10^-3 (6.59x10^-4)	8.29x10^-3 (6.34x10^-4)
Cerebellum	1.17x10^-1 (9.48x10^-3)	1.19x10^-1 (8.94x10^-3)	1.18x10^-1 (1.35x10^-2)	1.23x10^-1 (1.19x10^-2)
Hippocampus	8.20x10^-3 (5.78x10^-4)	8.66x10^-3 (7.55x10^-4)	8.22x10^-3 (9.93x10^-4)	7.84x10^-3 (1.17x10^-3)
Pallidum	4.13x10^-3 (2.32x10^-4)	3.94x10^-3 (4.67x10^-4)	3.78x10^-3 (6.03x10^-4)	3.98x10^-3 (6.22x10^-4)
Putamen	1.11x10^-2 (1.11x10^-3)	1.14x10^-2 (9.41x10^-4)	1.00x10^-2 (9.84x10^-4)	1.13x10^-2 (1.40x10^-3)
Thalamus	1.60x10^-2 (1.26x10^-3)	1.59x10^-2 (9.39x10^-4)	1.57x10^-2 (1.76x10^-3)	1.60x10^-2 (1.81x10^-3)
Ventral Diencephalon	7.77x10^-3 (6.66x10^-4)	8.01x10^-3 (7.78x10^-4)	8.01x10^-3 (9.78x10^-4)	8.34x10^-3 (1.03x10^-3)

LH = left hemisphere
RH = right hemisphere

eTable 11 General linear model results: Subcortical volume

**Region**	**Comparison**	***p*-value**	***Bayes factor***
Accumbens	LC vs. LP	**.01**	1.37x10^-2
	RC vs. RP	**<.001**	1.84x10^-2
	LP vs. RP	.62	3.25
Amygdala	LC vs. LP	.38	3.90
	RC vs. RP	.52	2.99
	LP vs. RP	.22	1.15
Caudate	LC vs. LP	**.02**	1.79x10^-1
	RC vs. RP	.99	7.99
	LP vs. RP	**<.01**	3.28x10^-2
Cerebellum	LC vs. LP	.56	6.31
	RC vs. RP	.93	7.59
	LP vs. RP	.66	4.34
Hippocampus	LC vs. LP	.77	7.80
	RC vs. RP	**.01**	4.75x10^-2
	LP vs. RP	.62	3.84
Pallidum	LC vs. LP	**<.001**	9.38x10^-3
	RC vs. RP	.93	6.07
	LP vs. RP	.22	1.06
Putamen	LC vs. LP	**<.001**	6.32x10^-4
	RC vs. RP	.93	6.99
	LP vs. RP	**<.01**	1.49x10^-3

Thalamus	LC vs. LP	.99	8.37
	RC vs. RP	.93	7.37
	LP vs. RP	.97	5.65
Ventral diencephalon	LC vs. LP	.55	5.39
	RC vs. RP	.99	8.00
	LP vs. RP	.91	5.43

LC = left hemisphere of controls
LP = left hemisphere of patients
RC = right hemisphere of controls
RP = right hemisphere of patients
p-values less than .05 are bolded.

Supplementary Material References

1. Fox J, Weisberg S. *An {R} Companion to Applied Regression*. 3rd ed. Thousand Oaks, CA: Sage; 2019.

2. Signorell A. *DescTools: Tools for Descriptive Statistics*.; 2021.

3. Bates D, Mächler M, Bolker B, Walker S. Fitting linear mixed-effects models using lme4. *J Stat Softw*. 2015;67(1):1-48. doi:10.18637/jss.v067.i01

4. Fortin J-P. *NeuroCombat: Harmonization of Multi-Site Imaging Data with ComBat*.; 2021.

5. Revelle W. *Psych: Procedures for Personality and Psychological Research*. Evanston, Illinois, USA: Northwestern Unviersity; 2021.

6. Wickham H. *Stringr: Simple, Consistent Wrappers for Common String Operations*.; 2019.

7. Wickham H, Averick M, Bryan J, et al. Welcome to the tidyverse. *JOSS*. 2019;4(43):1686. doi:10.21105/joss.01686
